# Forward stimulated Brillouin scattering and opto-mechanical non-reciprocity in standard polarization maintaining fibres

**DOI:** 10.1038/s41377-021-00557-y

**Published:** 2021-06-07

**Authors:** Gil Bashan, Hilel Hagai Diamandi, Yosef London, Kavita Sharma, Keren Shemer, Elad Zehavi, Avi Zadok

**Affiliations:** Faculty of Engineering and Institute for Nano-Technology and Advanced Materials, Bar-Ilan University, Ramat-Gan, 5290002 Israel

**Keywords:** Optical physics, Photoacoustics

## Abstract

Opto-mechanical interactions in guided wave media are drawing great interest in fundamental research and applications. Forward stimulated Brillouin scattering, in particular, is widely investigated in optical fibres and photonic integrated circuits. In this work, we report a comprehensive study of forward stimulated Brillouin scattering over standard, panda-type polarization maintaining fibres. We distinguish between intra-polarization scattering, in which two pump tones are co-polarized along one principal axis, and inter-polarization processes driven by orthogonally polarized pump waves. Both processes are quantified in analysis, calculations and experiment. Inter-modal scattering, in particular, introduces cross-polarization switching of probe waves that is non-reciprocal. Switching takes place in multiple wavelength windows. The results provide a first demonstration of opto-mechanical non-reciprocity of forward scatter in standard fibre. The inter-polarization process is applicable to distributed sensors of media outside the cladding and coating boundaries, where light cannot reach. The process may be scaled towards forward Brillouin lasers, optical isolators and circulators and narrowband microwave-photonic filters over longer sections of off-the-shelf polarization maintaining fibres.

## Introduction

Stimulated Brillouin scattering (SBS) is a nonlinear opto-mechanical interaction that couples between optical field components and acoustic waves that propagate in a common medium^1,2^. SBS supports narrowband oscillations of optical and acoustic waves^3–7^, all-optical and microwave-photonic signal processing^8–11^, spatially distributed sensing of temperature, strain, geometry and refractive index^12–14^, and more. SBS in optical fibres has been studied extensively since the 1970s^2^. The process may take place in the forward or backward directions^2,15^, with the two optical tones either co-propagating or counter-propagating. The backward effect is more widely known and employed in narrowband laser sources^3–5,16^, optical fibre sensors^12–14,17^ and microwave-photonic filters^18–21^. Forward SBS in fibre draws increasing interest in recent years, towards the analysis of media outside the cladding and coating^22–25^.

In the majority of scenarios, the two optical waves that take part in SBS propagate in the same guided optical mode. The acoustic waves involved in such intra-modal SBS in standard fibres take up specific characteristics: They are either purely axial (longitudinal) in the backwards SBS case^2^, or nearly entirely transverse for forward SBS^15,26,27^. Inter-modal SBS processes are also possible, when the two optical fields are guided in different spatial modes. Landmark demonstrations of inter-modal forward SBS have been reported in integrated photonic circuits in aluminium nitride^28^ and suspended silicon membranes^29–33^, leading to ultra-narrowband laser sources^29,32^ and microwave-photonic filters^31^. The acoustic axial wavenumber in inter-modal forward SBS is much larger than those of intra-modal processes^28–33^. This property gives rise to non-reciprocal propagation effects that are out of reach with intra-modal forward SBS^28,29,33^, and also removes the symmetry between scattering to the upper and lower probe sidebands^28–33^. Inter-modal Brillouin scattering is also possible in the backward direction^34^: the process has been demonstrated between the single-core mode and cladding modes of standard fibres, and is used in distributed sensing of refractive index outside the cladding^34^.

SBS processes are strongly dependent on the polarization of light^35–37^. In most fibre settings, the stimulation of acoustic waves is optimal when the two optical tones are co-polarized and the effect vanishes when their states of polarizations are orthogonal^36^. However, this restriction is not prohibitive: forward SBS involving two orthogonally polarized optical fields has been demonstrated and used in photonic crystal and nano-structured fibres^38–41^. Such inter-polarization processes represent a specific class of inter-modal scattering. However, this possibility has not yet been explored in standard fibres.

Polarization-maintaining (PM) fibres are being used in many studies of backward SBS. PM fibres support coupling between two intra-modal backward SBS processes, in so-called Brillouin dynamic gratings (BDGs)^42–47^: two counter-propagating pump tones polarized in one principal axis stimulate an acoustic wave and the same wave reflects probe light of the orthogonal polarization. BDGs are applied in distributed sensing^43^, microwave photonics^44^ and all-optical signal processing^45^. Similar processes were demonstrated with pumps and probe fields in two different guided modes of a few-order-mode fibre^46^. It is noteworthy, however, that BDGs do not constitute inter-modal scattering, as orthogonally polarized optical fields are not coupled directly by acoustic waves. Spontaneous Brillouin scattering in the forward direction over PM fibres has been studied in a series of papers in the 1990s^48–50^. To the best of our knowledge, the corresponding stimulated processes have not been investigated.

In this work we report a comprehensive first study of forward SBS in a standard, commercially available panda-type PM fibre. Due to the presence of strain rods, the fibre supports large number of guided acoustic modes with complex transverse profiles. These modes give rise to forward SBS spectra that are far more complex than those of standard single-mode fibres (SMFs). Nevertheless, the processes are modelled and predicted in very good agreement with experiment. Due to the lack of radial symmetry of the elastic properties in the fibre cross-section, intra-modal forward SBS processes along the fast and slow axes exhibit different spectra. We show that intra-modal forward SBS also induces cross-phase modulation (XPM) between the two principal axes. This effect represents a forward SBS equivalent to the BDGs of backward scattering in PM fibres^42–47^.

Moreover, forward SBS driven by two orthogonally polarized optical pump tones is demonstrated for the first time in a standard fibre. The acoustic waves stimulated in these inter-modal processes include axial wavenumbers that are 1000 times larger than those of intra-modal forward scattering. Inter-modal forward SBS gives rise to cross-polarization switching of probe waves that are counter-propagating with respect to the pumps. This characteristic could prove to be extremely useful in sensors applications. Preliminary measurements of the inter-polarization forward SBS spectrum with air or ethanol outside the cladding show large differences. Forward scattering events are inherently difficult to localize and the monitoring of a counter-propagating probe wave may address the challenges associated with its spatially distributed analysis^23–25^. Lastly, the cross-polarization coupling is non-reciprocal: a probe wave of the same frequency that is co-propagating with the pump fields is unaffected. Non-reciprocity can be induced in several probe wavelength windows using the same pump wavelength, through a multitude of guided acoustic modes. When scaled to sufficiently long sections of an off-the-shelf PM fibre, the inter-modal process can provide the building blocks for opto-mechanical isolators and circulators, forward Brillouin lasers and ultra-narrow microwave-photonic filters.

## Results

### Principle of operation: forward SBS processes in polarization-maintaining fibres

**Fig. 1:**
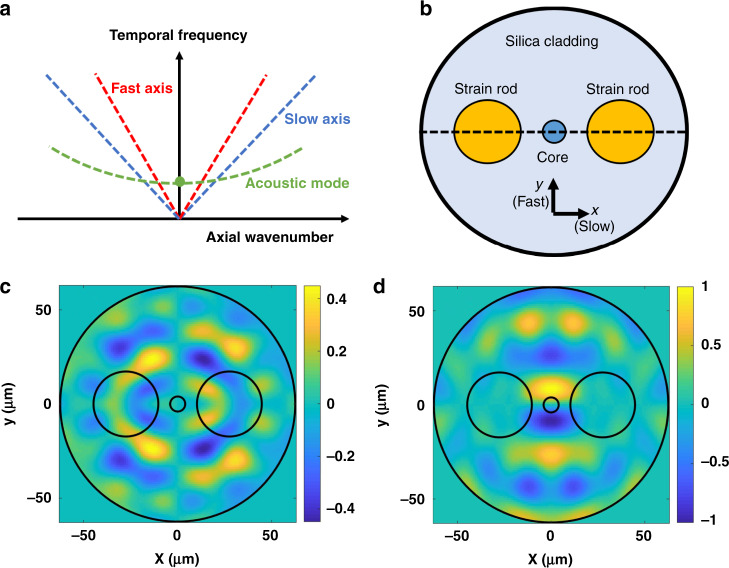
Polarization-maintaining fibres. **a** Schematic illustration of the dispersion relations between temporal frequency and axial wavenumber for light guided in the fast axis (red) and slow axis (blue), and for a guided acoustic mode (green). The circular green marker denotes the modal cut-off frequency. **b** Schematic cross-section of a panda-type polarization-maintaining fibre, with two B_2_O_3_-doped silica strain rods embedded in the pure silica cladding. The centres of the rods are placed at equal distances from the fibre axis in the $\pm {\hat{\boldsymbol x}}$ directions. Thermal expansion of the rods during fibre production induces permanent strain and birefringence in the fibre, with ${\hat{\boldsymbol x}}$ being the slow axis. **c**, **d** Calculated normalized transverse profiles of material displacement in one guided acoustic mode, with a cut-off frequency of 175 MHz. **c**, **d** Displacement components in the ${\hat{\boldsymbol x}}$ and ${\hat{\boldsymbol y}}$ directions, respectively. Black circles denote the boundaries of the cladding, strain rods, and core

Figure 1a illustrates the dispersion relations between optical frequency and axial wavenumber of the single optical mode in the slow (${\hat{\boldsymbol x}}$) and fast (${\hat{\boldsymbol y}}$) axes of a PM fibre. We disregard chromatic dispersion. The slopes of the dispersion relations are $c/n_{s,f}$ for the slow and fast axes, respectively, where *c* is the speed of light in vacuum and $n_{s,f}$ are the corresponding refractive indices. The difference between the two indices is typically in the fourth decimal point^51^. The figure also demonstrates the dispersion relation between frequency ${{\Omega }}$ and axial wavenumber $q_m$ of an acoustic mode *m* that is guided by the fibre cross-section. Each mode is characterized by a cut-off frequency, ${{\Omega }}_m$, below which it may not propagate^15,26,27^. This work addresses modes with cut-off frequencies in the hundreds of MHz range. Close to cut-off, the axial phase velocity of the acoustic wave, ${{\Omega }}/q_m$, approaches infinity and may equal the optical phase velocities $c/n_{s,f}$^15,26,27^.

Figure 1b shows an illustration of the cross-section of a panda-type PM fibre used in this work. It includes two strain rods of silica doped with B_2_O_3_. Expansion of the rods during fibre production induces permanent strain and photo-elastic birefringence in the silica fibre. Due to the lack of radial symmetry, the PM fibre cross-section supports large number of guided acoustic modes with complicated transverse profiles. Figure 1c, d show an example of the calculated transverse profiles of normalized material displacement in the ${\hat{\boldsymbol x}}$ and ${\hat{\boldsymbol y}}$ directions for one acoustic mode, with a cut-off frequency of 175 MHz. For detailed calculation of modal profiles and cut-off frequencies, see Supplementary Information 1.

Two co-propagating optical waves and a guided acoustic wave can be coupled in a forward SBS process^15,26,27^. The optical fields give rise to an electro-strictive driving force that may stimulate acoustic oscillations. The acoustic wave, in turn, may scatter and modulate the two optical waves that initiate the process, as well as additional optical probe fields^15,26,27^. Most processes described in this work involve more than three waves. We therefore refer to the two optical fields that stimulate the acoustic waves as pump tones, similar to the terminology used in BDG literature^42–47^. Forward SBS processes take place on two conditions as follows: the first one is an agreement between transverse profiles^15,26,27^. The spatial profile of the electro-strictive driving force must sufficiently overlap with that of the acoustic modal displacement^15,26,27^. The same holds true for the transverse profiles of acoustically induced photo-elastic perturbations across the fibre and the optical mode^15,26,27^. Spatial overlap considerations are formulated in Supplementary Information 2 and 3. Their calculations are generally more complicated than those of forward SBS in SMF, due to the lack of radial symmetry in the panda PM fibre structure.

**Fig. 2:**
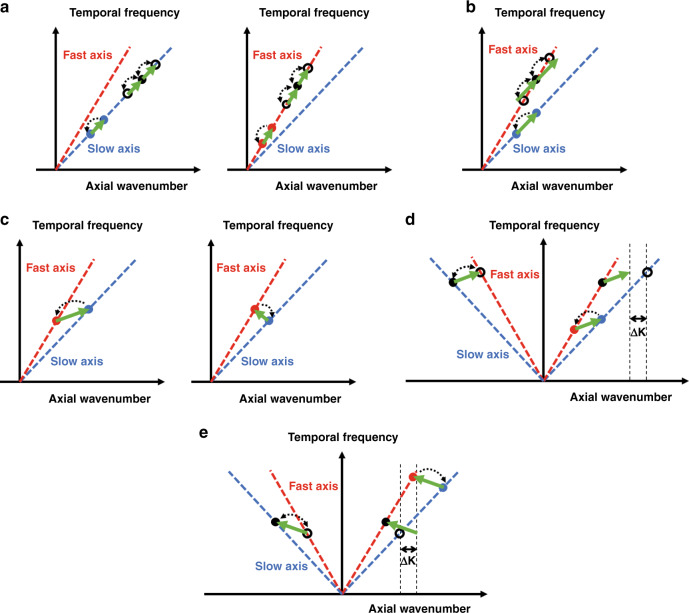
Forward stimulated Brillouin scattering processes in polarization-maintaining fibres. **a** Schematic illustration of intra-modal forward SBS along the slow axis (left) or fast axis (right). Two pump tones (full blue circular markers for the slow axis process on the left, full red markers for the fast axis process on the right) stimulate a guided acoustic wave (green arrow). The same acoustic wave modulates a co-polarized, co-propagating probe wave (full black circle), and generates two sidebands (empty circular markers). Dashed black arrows denote acoustically induced coupling. **b** Cross-polarization phase modulation through intra-modal forward SBS. Two pump tones in the slow axis generate an acoustic wave. The acoustic wave, in turn, generates two sidebands of a probe wave polarized along the fast axis. The process is associated with a nonzero wavenumber mismatch, due to the PM fibre birefringence. However, the mismatch is small and has negligible effect over metre-long fibres. An equivalent process may take place with the pump tones along the fast axis and a probe wave aligned with the slow axis instead. **c** Inter-modal forward SBS. Two pump tones of orthogonal polarizations stimulate an acoustic wave. The axial wavenumber of the acoustic wave is much larger than those of intra-modal forward SBS. When the higher-frequency pump was polarized in the slow axis, the stimulated acoustic wave is co-propagating with the two pumps (left). If the higher-frequency pump is in the fast axis instead, the acoustic wave is counter-propagating (right). **d** Non-reciprocal cross-polarization coupling of probe waves due to inter-modal forward SBS. The acoustic wave may couple a probe wave of specific frequency that is counter-propagating with respect to the pumps to the orthogonal polarization. Cross-polarization coupling of probe light with the same frequency is prohibited in the forward direction, due to wavenumber mismatch (noted as $\Delta k$). The acoustic wave is co-propagating with the two pump tones (as in **c**, left). **e** Same as **d**, for an inter-polarization forward SBS process in which the acoustic wave is counter-propagating with respect to the two pumps (see also **c**, right)

The second necessary condition is axial wavenumber matching between the pair of optical tones and the acoustic wave involved^15,26,27^. This condition can be met when the optical frequencies of the two pump waves are $\omega _{\mathrm{p}} \pm \frac{1}{2}{{\Omega }}$, where $\omega _{\mathrm{p}}$ is a central optical frequency and ${{\Omega }}$ represents a radiofrequency detuning that is close to the modal cut-off ${{\Omega }}_m$^15,26,27^. In a PM fibre, wavenumber matching may be fulfilled through several different choices of the two optical polarizations. In intra-modal forward SBS, the two pump tones are co-polarized along either the slow or the fast principal axis (Fig. 2a). The transverse profile of the electro-strictive driving force induced by the pump waves is given by^15,26,27^ (Supplementary Information 2):

1 $$\mathop{f}\limits^{\rightharpoonup} \propto - |E_{\mathrm{T}}(r)|^2\left[ {(2a_1 + 4a_2)} \right]r\hat r \pm 2a_1(x\hat x - y\hat y)$$

here, $r$ is the transverse radial coordinate, $\hat r$ denotes the unit vector in the radial direction and $x$, $y$ are transverse coordinates in the $\hat x$ and $\hat y$ directions, respectively. Next, *a*_1_ = 0.66 and *a*_2_ = −1.19 denote electro-strictive parameters drawn from the photo-elastic tensor of silica^15,26,27^ (Supplementary information 1), and $E_{\mathrm{T}}\left( r \right)$ is the normalized transverse profile of the optical mode, which is radially symmetric. The driving force consists of a first term that is radially symmetric and independent of pumps polarization, and a second term that varies with the choice of axis: the ± signs in Eq. (1) correspond to pump tones along the ${\hat{\boldsymbol y}}$ or ${\hat{\boldsymbol x}}$ axes, respectively. The overall electro-strictive driving force is therefore polarization dependent. The pump tones stimulate the oscillation of a co-propagating acoustic wave in mode $m$. The axial wavenumber of the acoustic waves is given by $q_m \approx {{\Omega }}n_{s,f}/c$, on the order of 1 rad m^−1^. The axial wavenumber is orders of magnitude smaller than those of bulk acoustic waves in the cladding or strain rods. Therefore, the acoustic wave vectors are almost entirely transverse. The group velocity in the axial direction is vanishingly small; hence, acoustic energy does not propagate along the fibre. The stimulation is accompanied by an exchange of power between the two pump waves^15,26,27^.

Intra-modal forward SBS may also affect the propagation of an optical probe wave of frequency $\omega _{\mathrm{sig}}\, \ne \,\omega _{\mathrm{p}}$ that is co-polarized and co-propagating with the pump tones. The probe is scattered by the acoustic waves to lower and upper sidebands of frequencies $\omega _{\mathrm{sig}} \pm {{\Omega }}$^15,26,27^ (Fig. 2a). In the absence of chromatic dispersion, the scattering of a co-polarized probe wave is inherently wavenumber matched. The generation of sidebands represents phase modulation of the probe^52–54^. This process is entirely equivalent to forward SBS in SMF, which was studied extensively^52–54^. The intra-polarization XPM between pumps and probe adds on top of Kerr effect contributions^55^. The process is quantified by a modal opto-mechanical coefficient $\gamma _m\left( {{\Omega }} \right)$, in units of W^−1^ m^−1^ (Supplementary Information 2 and 3^56,57^):

2 $$\gamma _m\left( {{\Omega }} \right) = \frac{{k_0Q_{\mathrm{ES}}^{\left( m \right)}Q_{\mathrm{PE}}^{\left( m \right)}}}{{8n^2c\rho _1}}H_m\left( {{\Omega }} \right)$$

here, $k_0$ is the vacuum wavenumber at the frequency of the probe wave, $\rho _1$ denotes the density of silica, $n \approx n_{s,f}$ and $H_m\left( {{\Omega }} \right) = 1/\left( {{{\Omega }}_m^2 - {{\Omega }}^2 - j{{\Gamma }}_m{{\Omega }}} \right)$. ${{\Gamma }}_m$ denotes the modal linewidth, which also signifies the decay rate of acoustic energy^22–25^. In most of this work, we consider only bare fibres that are stripped off their protective coatings and kept in air. In this case, there is no loss of acoustic energy to the fibre surroundings and ${{\Gamma }}_m$ is determined entirely by internal acoustic dissipation in the silica cladding and strain rods. The linewidths are fitted based on experiments (see ‘Methods’, as well as Supplementary Information 2). Also in Eq. (1), $Q_{\mathrm{ES}}^{\left( m \right)}$ and $Q_{\mathrm{PE}}^{\left( m \right)}$ denote overlap integrals of electro-strictive stimulation and photo-elastic scattering, respectively^15,26,27^ (see Supplementary Information 2). It is worth noting that $Q_{\mathrm{ES}}^{\left( m \right)}$ depends on the choice of pump waves polarization—slow or fast axes—and $Q_{\mathrm{PE}}^{\left( m \right)}$ varies with the alignment of the probe wave polarization. Therefore, intra-modal forward SBS processes along the two principal axes are not the same. The opto-mechanical coefficients obtain their largest magnitudes at the modal cut-off:

3 $$\gamma _{0m} = \gamma _m\left( {{{\Omega }}_m} \right) = j\frac{{k_0Q_{\mathrm{ES}}^{\left( m \right)}Q_{\mathrm{PE}}^{\left( m \right)}}}{{8n^2c\rho _1{{\Gamma }}_m{{\Omega }}_m}}$$

The overall coefficient at a given frequency is given by the sum over all modes: $\gamma \left( {{\Omega }} \right) = \mathop {\sum }\limits_m \gamma _m\left( {{\Omega }} \right)$. The magnitude of the probe phase modulation at the output of a fibre of length $L$ equals^22,52^:

4 $$\Delta \tilde \varphi \left( {{\Omega }} \right) = \gamma \left( {{\Omega }} \right)\tilde P\left( {{\Omega }} \right)L$$

In Eq. (4), $\tilde P\left( {{\Omega }} \right)$ represents the radiofrequency Fourier component of the optical power of the two pump waves combined^22,52^.

Intra-modal forward SBS in PM fibres can also give rise to inter-polarization XPM, between pump light in one principal axis and a probe wave in the orthogonal one. The process is illustrated in Fig. 2b. The generation of sidebands for an orthogonal probe wave is subject to a nonzero wavenumber mismatch, due to the PM fibre birefringence: $\Delta k_{\mathrm{mis}} = \pm {{\Omega }}\left( {n_s - n_f} \right)/c$. The mismatch, however, is very small: on the order of only ±0.001 rad m^−1^. Therefore, this wavenumber mismatch has little effect on the modulation of the orthogonal probe wave over the metre-long fibres used in this work. The acoustic wave effectively couples between two separate intra-modal forward SBS processes, similar to BDGs in backward scattering over PM fibres^42–47^. In this case as well, the inter-polarization XPM due to SBS adds up with that of the Kerr effect^55^.

Intra-modal scattering is not the only possibility for forward SBS interactions in PM fibres. Figure 2c illustrates inter-modal scattering, in which the polarizations of the two pump tones are orthogonal. The two orthogonal pump waves induce an electro-strictive driving force of frequency ${{\Omega }}$^38^. The transverse profile of the driving force in this case is given by^38^ (see Supplementary Information 3):

5 $$\mathop{f}\limits^{\rightharpoonup} \propto 2a_1\left| {E_{\mathrm{T}}\left( r \right)} \right|^2\left( {b_{45}\hat e_{45} - b_{ - 45}\hat e_{ - 45}} \right)$$

here, $\hat e_{ \pm 45} = \left( {1/\sqrt 2 } \right)\left( {\hat x \pm \hat y} \right)$ are unit Jones vectors of linear polarizations oriented at ±45° with respect to the principal axes and the projections $b_{ \pm 45}$ are given by $\left( {1/\sqrt 2 } \right)\left( {x \pm y} \right)$. Compared with intra-modal processes, the inter-modal electro-strictive driving force does not include a radially symmetric term and its remaining component is rotated by 45°.

When the higher-frequency pump wave is polarized in the slow axis, the stimulated acoustic wave is co-propagating with the two pumps (Fig. 2c, left). If the higher-frequency pump is aligned with the fast axis instead, the acoustic wave would be counter-propagating (Fig. 2c, right). The stimulation of the acoustic wave is associated with the coupling of optical power between the two pump tones, similar to other SBS processes. The coupling of power is governed by $2{\mathrm{Im}}\left[ {\gamma \left( {{\Omega }} \right)} \right]$ (see Supplementary Information 3). The acoustic wavevector in inter-modal forward SBS includes an axial component $q_m \approx \pm \omega _{\mathrm{p}}\left( {n_s - n_f} \right)/c$. In standard PM fibres, this component is on the order of ±1000 rad m^−1^: three orders of magnitude larger than the intra-modal forward SBS case. The axial wavenumber $q_m$ is still orders of magnitude smaller than those of bulk acoustic waves in the silica cladding or the strain rods. The axial group velocity and axial displacement both remain very small, so that the stimulated acoustic waves are still predominantly transverse. Nevertheless, their axial wavenumber is sufficiently large to give rise to non-reciprocal polarization switching of probe waves, as discussed next. The larger axial wavenumber also removes the symmetry between the scattering of probe waves to the upper and lower sidebands^28–33,38–41^. Such removal of symmetry leads to intensity modulation of probe waves rather than pure phase modulation^28–33,38–41^.

Figure 2d illustrates a probe wave that is counter-propagating with respect to the pumps in the negative $- {\hat{\boldsymbol z}}$ direction with frequency $\omega _{\mathrm{sig}}$. The probe is polarized in the slow ${\hat{\boldsymbol x}}$ axis. We consider the case of inter-modal forward SBS leading to a co-propagating stimulated acoustic wave (as in Fig. 2c, left). The acoustic wave may scatter the probe into a component of frequency $\omega _{\mathrm{sig}} + {{\Omega }}$ in the orthogonal ${\hat{\boldsymbol y}}$ polarization, which is also propagating in the $- {\hat{\boldsymbol z}}$ direction. The process is wavenumber matched when the difference in optical frequencies between probe and pumps, $\omega _{\mathrm{sig}} - \omega _{\mathrm{p}}$, is adjusted to a specific value (Supplementary Information 3):

6 $$\Delta \omega _{\mathrm{opt}} = \left( {\omega _{\mathrm{sig}} - \omega _{\mathrm{p}}} \right) \approx \frac{{2n}}{{n_s - n_f}}{{\Omega }}$$

The cross-polarization coupling process is non-reciprocal. A probe wave co-propagating with the pumps in the $+ {\hat{\boldsymbol z}}$ direction with the same frequency $\omega _{\mathrm{p}} + \Delta \omega _{\mathrm{opt}}$ cannot couple to the orthogonal polarization (Fig. 2d). Such coupling is hindered by a wavenumber mismatch of $\Delta k_{\mathrm{mis}} \approx 2n{{\Omega }}/c$, on the order of 10 rad m^−1^ (see Supplementary Information 3). The efficiency of the process over several metres of fibre will be significantly degraded. In the case of inter-modal forward SBS leading to a counter-propagating acoustic wave (Fig. 2c, right), a ${\hat{\boldsymbol y}}$ polarized probe at frequency $\omega _{\mathrm{sig}}$ in the $- {\hat{\boldsymbol z}}$ direction could couple with an ${\hat{\boldsymbol x}}$ polarized wave component of frequency $\omega _{\mathrm{sig}} + {{\Omega }}$. In that case, wavenumber matching as is achieved when $\omega _{\mathrm{sig}} = \omega _{\mathrm{p}} - \Delta \omega _{\mathrm{opt}}$ (see Fig. 2e and Supplementary Information 3).

In panda-type PM fibres, the difference $\Delta \omega _{\mathrm{opt}}$ of Eq. (6) is in the THz range, corresponding to several nanometres difference in wavelengths between pumps and probe. The inter-modal forward SBS spectrum comprises multiple resonance frequencies. Therefore, cross-polarization coupling of probe light may take place in several wavelength windows for the same pumps frequency $\omega _{\mathrm{p}}$, through different choices of ${{\Omega }}$. The analysis suggests that inter-modal forward SBS non-reciprocity, successfully demonstrated in specialty nano-structured fibres and photonic integrated circuits^28–33,38–41^, is directly applicable in standard, off-the-shelf optical fibres as well.

### Measurements and numerical calculations

**Fig. 3:**
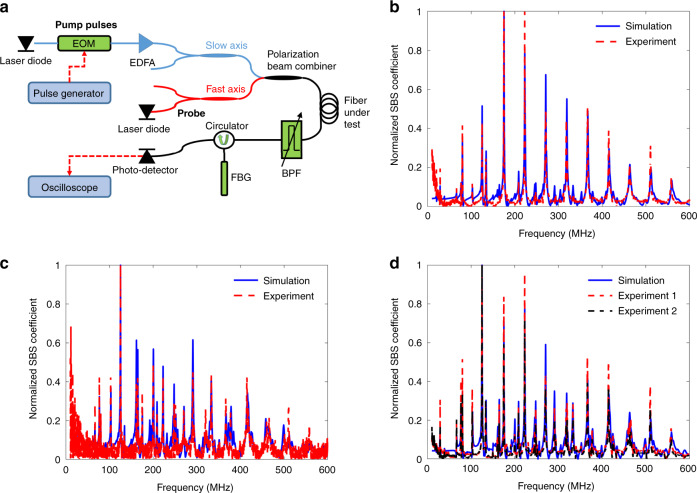
Measurements and calculations of intra-modal forward SBS in polarization-maintaining fibres. **a** Schematic illustration of the experimental setup. EDFA: erbium-doped fibre amplifier; EOM: electro-optic amplitude modulator; FBG: fibre Bragg grating; BPF: optical bandpass filter. In the illustration, pump pulses are launched along the slow axis and probe light is coupled to the fast axis. Both pump and probe may be connected to either the slow or fast axes, as necessary. **b** Measured (dashed red) and calculated (solid blue) normalized absolute value of the intra-modal forward SBS coefficient. Acoustic waves were stimulated by pump pulses polarized in the slow axis. Phase modulation of a probe wave co-polarized along the slow axis was observed. Very good agreement is found between model and experiment. **c** Same as **b**, with the pump pulses and probe wave moved to the fast axis instead. The spectrum of intra-modal forward SBS is different between the two axes. **d** Measured (dashed red and dashed black) and calculated (solid blue) normalized absolute value of the coefficient of cross-polarization phase modulation due to intra-modal forward SBS. In the red trace, pump pulses were launched along the fast axis and the probe was aligned with the slow axis. In the black trace, the two polarizations were switched. The spectra in both cases are identical, in agreement with expectations and calculations

The experimental setup used in measurements of intra-modal forward SBS over PM fibres is illustrated in Fig. 3a^22,52,57^. A first laser diode of 1543 nm wavelength was used as the source of pump light. The pump wave was modulated into repeating pulses of 1.5 ns duration and 5 μs period using an electro-optic amplitude modulator. The duration of pulses was chosen to match the bandwidth of forward SBS processes. The pump pulses were then amplified by an erbium-doped fibre amplifier (EDFA) to an average power of 300 mW and coupled to the input end of a PM fibre under test, in either the slow or fast axis. The power was limited by the available amplifier. The fibre was 6 m long. It was stripped of its protective polymer coating and kept in air. The instantaneous pulse shape of the pump wave was recorded and its Fourier transform $\tilde P\left( {{\Omega }} \right)$ was calculated offline for later use in data analysis (see below).

A continuous probe wave from a second laser diode of 1550 nm wavelength and 10 mW power was also coupled into the fibre under test, in the same direction of propagation. The probe wave could also be aligned with the slow or fast axes as necessary. An optical bandpass filter at the output end of the fibre was tuned to select the probe wavelength only and reject the pump pulses. The output probe wave was then reflected form a fibre Bragg grating (FBG) with 0.02 nm bandwidth and maximum reflectivity of 90%. The probe frequency was precisely adjusted to a spectral slope of the FBG reflectivity spectrum. Phase modulation of the probe wave due to forward SBS was thereby converted to an intensity signal. The reflected probe wave was detected by a broadband photo-receiver (responsivity of 27 VW^−1^, 15 ps rise time). The detector voltage $V\left( t \right)$ was proportional to the instantaneous frequency modulation of the probe wave: ${\mathrm{d}}\left[ {\Delta \varphi \left( t \right)} \right]/{\mathrm{d}}t$, where $\Delta \varphi \left( t \right)$ is the temporal phase modulation and $t$ stands for time. The detector voltage was sampled by a real-time digitizing oscilloscope for further offline processing.

As noted earlier, XPM of the probe wave takes place through forward SBS and the Kerr effect as well^55^. However, the contribution of the instantaneous Kerr effect vanishes as soon as the pump pulse ends, whereas the stimulated acoustic waves continue to oscillate for hundreds of nanoseconds^22–25^. The sampled traces $V\left( t \right)$ were gated to remove the first 10 ns, so that the contribution of the Kerr effect was eliminated. The normalized intra-modal forward SBS spectrum of the fibre under test was estimated based on the Fourier transform $\tilde V\left( {{\Omega }} \right)$ of the gated $V\left( t \right)$ trace:

7 $$\left| {\gamma \left( {{\Omega }} \right)} \right| \propto \left| {\tilde V\left( {{\Omega }} \right)} \right|/\left[ {\left| {\tilde P\left( {{\Omega }} \right)} \right|{{\Omega }}} \right]$$

Figure 3b, c show the measured and calculated normalized coefficients of intra-modal forward SBS XPM along the slow and fast axes, respectively. Both spectra consist of a large number of resonances, with overall complex shapes. The measured spectra are in excellent agreement with calculations (see Supplementary Information 1 and 2). As expected, the forward SBS processes along the two principal axes are different: the spectrum for slow axis alignment is closer to that of SMF^15^, whereas fast axis polarization leads to less regular spectrum with larger number of resonance frequencies. The highest calculated coefficient $\left| {\gamma \left( {{\Omega }} \right)} \right|$ is 5.3 W^−1^ km^−1^ along the slow axis (175 MHz) and 3.5 W^−1^ km^−1^ for the fast axis (125 MHz). For comparison, the largest $\left| {\gamma \left( {{\Omega }} \right)} \right|$ of forward SBS in bare SMF is on the order of 20 W^−1^ km^−1^^57^. Figure 3d presents the measured and calculated normalized coefficient of cross-polarization phase modulation due to intra-modal forward SBS. Here too, the experiment is well supported by calculations. Two measured traces are shown: one with pump pulses along the slow axis and a probe wave in the fast axis, and another where the two polarizations were switched. The two spectra are identical, in agreement with calculations and previous analyses and observations^37,58^. The largest calculated coefficient $\left| {\gamma \left( {{\Omega }} \right)} \right|$ for the process is 3.1 W^−1^ km^−1^, at 125 MHz.

**Fig. 4:**
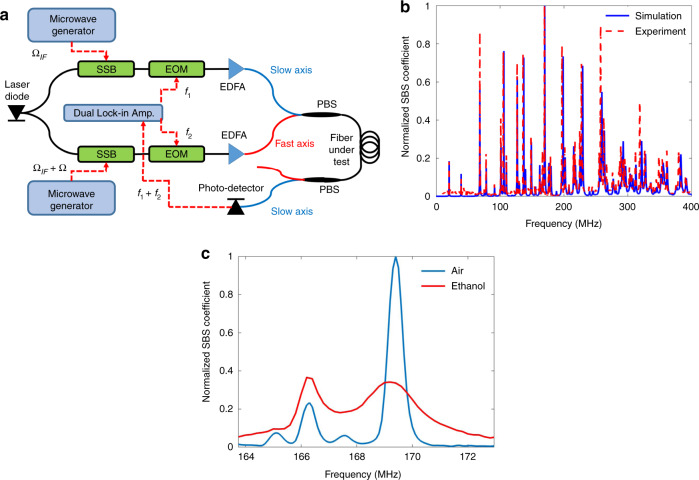
Measurements and calculations of inter-modal forward SBS in polarization-maintaining fibres. **a** Schematic illustration of the experimental setup. EDFA: erbium-doped fibre amplifier; SSB: single-sideband electro-optic modulator; EOM: electro-optic amplitude modulator; PBS: polarization beam splitter. **b** Measured (dashed red) and calculated (solid blue) normalized coefficient of the coupling of power between the two pump tones in inter-modal forward SBS. Agreement between model and measurement is very good. **c** Measured normalized coefficient of the coupling of power between the pump tones with air (blue) and ethanol (red) outside the bare fibre cladding. Modal linewidths are broader with ethanol outside the cladding due to acoustic dissipation^22–25^

The experimental setup for inter-polarization forward SBS is illustrated in Fig. 4a. Light from a laser diode is split into two paths. The optical wave in one branch was upshifted by a fixed intermediate frequency offset ${{\Omega }}_{\mathrm{IF}}$ of 2*π* × 10 GHz using a single-sideband (SSB) electro-optic modulator. A sine wave of radiofrequency $f_1$ of 2*π* × 40 kHz from one output port of a dual lock-in amplifier was superimposed on the modulating waveform using an electro-optic amplitude modulator. Light in the first branch was then amplified by an EDFA to 500 mW power and launched into a bare 20 m-long panda PM fibre under test along the slow axis through a polarization beam splitter. Light in the second branch was upshifted by a variable frequency ${{\Omega }}_{\mathrm{IF}} + {{\Omega }}$ using a second SSB modulator, amplitude modulated by a sine wave of frequency $f_2$ = 2*π* × 50 kHz from a second output port of the same lock-in amplifier in another electro-optic amplitude modulator, amplified in a second EDFA to 500 mW power and launched along the fast axis of the fibre under test. Leakage between principal axes in the polarization beam splitter was below −25 dB: too weak to stimulate intra-modal forward SBS. A second polarization beam splitter at the output end of the fibre routed the slow-axis wave component to a photo-receiver.

The detected voltage was analysed by the lock-in amplifier input. Coupling of optical power between the two tones due to forward SBS at frequency ${{\Omega }}$ was identified through oscillations of the detected voltage at the sum radiofrequency $f_1 + f_2$^59^. The coupling scales with the imaginary part of the inter-modal forward SBS coefficient: $2{\mathrm{Im}}\left[ {\gamma \left( {{\Omega }} \right)} \right]$ (see Supplementary Information 3). Figure 4b shows the measured and calculated normalized coefficient. The results demonstrate that forward SBS takes place between orthogonally polarized pump waves. Once more, agreement between model and measurements is very good. As anticipated, the spectrum is different from those of intra-modal forward SBS processes in the same fibre (Fig. 3). The largest measured coefficient $2{\mathrm{Im}}\left[ {\gamma \left( {{\Omega }} \right)} \right]$ for inter-modal forward SBS was 1.2 ± 0.2 W^−1^ km^−1^, at 169 MHz (see ‘Methods’ for calibration details). The measurement is in agreement with the corresponding calculated value of 1.4 W^−1^ km^−1^. Figure 4c shows the measured normalized coefficient of inter-polarization forward SBS with air (blue) or ethanol (red) outside the bare fibre cladding. The two spectra are markedly different. The dissipation of acoustic waves to the outside liquid manifests in broader modal linewidths^22–25^. Inter-polarization scattering provides unique opportunities for forward SBS fibre sensing through polarization switching of probe waves (see ‘Discussion’ section).

**Fig. 5:**
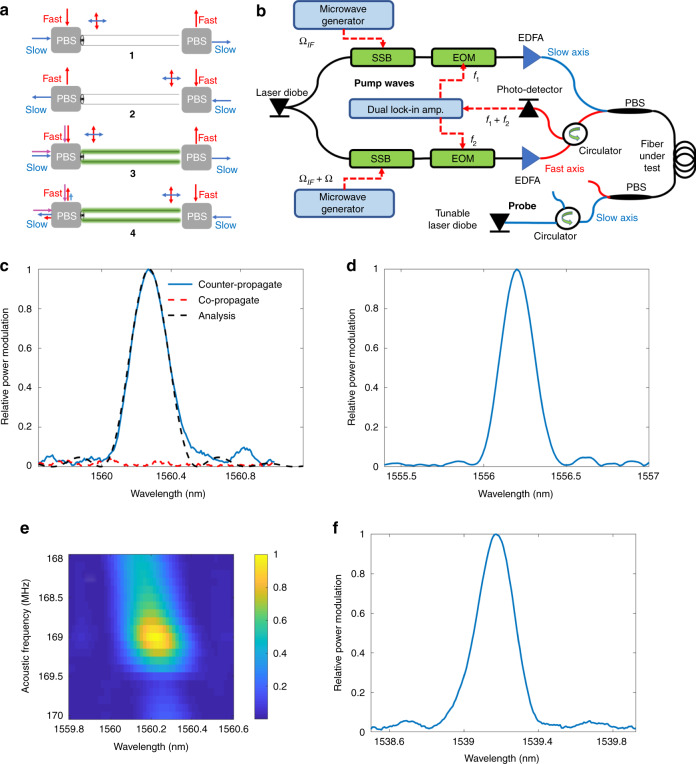
Non-reciprocal cross-polarization coupling of probe waves in inter-modal forward SBS over PM fibre. **a** Schematic illustration of the principle of operation. With no forward SBS pumps, probe signals in the slow (blue) or fast (red) axes do not couple to the orthogonal polarization states. No polarization switching takes place in both directions of propagation (sketches 1 and 2). When pump tones are propagated from the left to right (purple arrows, sketches 3 and 4), non-reciprocal coupling of probe light from one principal axis to the other may take place, depending on the choice of frequencies. Probe light that is counter-propagating with respect to the pumps can be partially switched to the orthogonal state (sketch 4). On the other hand, probe waves of the same frequencies that co-propagate with the pump tones do not exhibit polarization coupling (sketch 3). **b** Schematic illustration of the experimental setup. EDFA: erbium-doped fibre amplifier; SSB: single-sideband electro-optic modulator; EOM: electro-optic amplitude modulator; PBS: polarization beam splitter. **c** Normalized lock-in voltage of probe wave modulation, as a function of probe wavelength. Blue solid trace, measurement when the probe wave is counter-propagated with respect to two orthogonally polarized forward SBS pumps. The difference in optical frequencies between the two pump waves is 169 MHz. The higher-frequency pump wave is polarized along the fast axis and the lower-frequency one along the slow axis. The probe wave is launched along the slow axis. The acoustic wave stimulated in the inter-polarization forward SBS process leads to cross-polarization coupling of the counter-propagating probe wave to the fast axis. The obtained signal is maximal at a wavelength difference of 10.8 nm between pumps and probe, which corresponds to a birefringence of 3.8 × 10^-4^ refractive index units in the PM fibre. Black dashed trace, corresponding calculated normalized spectrum of probe cross-polarization coupling. Agreement between model and experiment is excellent. Red dashed trace, same measurement when the probe wave co-propagates with the pump tones in the same direction. No modulation is observed, as wavenumber mismatch inhibits the cross-polarization coupling of the probe in this case. The process is therefore non-reciprocal. The relative isolation between the two directions of propagation is at least 20 dB. **d** Same as the blue trace of **c**, with the difference between the optical frequencies of the pump tones changed to 105 MHz. Maximum modulation of the probe wave is obtained at a wavelength of 1556.2 nm, closer to that of the pumps, as expected. **e** Two-dimensional scan of the normalized magnitude of the probe wave modulation, as a function of both probe wavelength and the optical frequency difference between the two pump tones. **f** Same as the blue trace of **c**, with the polarizations of the two pump waves switched: the higher-frequency pump is polarized along the slow axis and the lower-frequency pump tone along the fast axis. In this case, maximum modulation of the output probe wave is observed at a shorter wavelength than that of the pumps

The cross-polarization switching of probe waves that are counter-propagating with respect to the pump tones was demonstrated experimentally. The process is illustrated in Fig. 5a and the setup is depicted in Fig. 5b. Two orthogonally polarized pump waves with a variable difference ${{\Omega }}$ between their optical frequencies and a central wavelength of 1550 ± 0.2 nm were prepared as described above. Once more, the two tones were amplitude modulated by lock-in signals of low radiofrequencies $f_{1,2}$ = 2*π* × 40 kHz and 2*π* × 50 kHz, respectively, and launched into the fibre under test in the $+ {\hat{\boldsymbol z}}$ direction. The two pumps stimulated acoustic waves at frequency ${{\Omega }}$ through an inter-polarization forward SBS process. The magnitude of the acoustic wave was modulated at the sum frequency $f_1 + f_2$. A continuous probe wave of tuneable wavelength $\lambda _s$ and 5 mW power was polarized along the slow axis and launched into the fibre under test from the opposite end, in the $- {\hat{\boldsymbol z}}$ direction. The output probe wave passed through a polarization beam splitter and the fast axis component was detected by a photo-receiver. An optical bandpass filter (not shown) blocked potential leakage of the pump waves from reaching the receiver. Cross-polarization coupling of the probe wave was quantified by the modulation of the detector voltage at the sum frequency $f_1 + f_2$.

Figure 5c (solid blue trace) shows the normalized intensity modulation of the probe wave at the fast axis output as a function of $\lambda _s$, with the difference between pump frequencies ${{\Omega }}$ set to 2*π* × 169 MHz. Maximum modulation is observed at a probe wavelength $\lambda _{\mathrm{max}}$ of 1560.2 nm. The measured spectrum is an excellent agreement with calculations (see Supplementary Information 3). The difference between pumps wavelength and $\lambda _{\mathrm{max}}$ suggests a birefringence $n_s - n_f$ of 3.8 × 10^−4^ refractive index units in the panda PM fibre (see Eq. (6)). This estimate is in good agreement with reference values^51^. The full width at half maximum of the cross-polarization coupling spectrum is 0.25 nm. Figure 5d shows the corresponding trace with ${{\Omega }}$ changed to 2*π* × 105 MHz. The wavelength $\lambda _{\mathrm{max}}$ in this case is modified to 1556.2 nm. The difference between the pumps wavelength and $\lambda _{\mathrm{max}}$ scales with ${{\Omega }}$, as anticipated.

Figure 5e presents a two-dimensional scan of the probe wave cross-polarization coupling as a function of both ${{\Omega }}$ and $\lambda _{\mathrm{s}}$, in the vicinity of a single peak of inter-polarization forward SBS. The dependence on ${{\Omega }}$ represents the efficiency of acoustic wave stimulation by the pump tones, whereas the $\lambda _{\mathrm{s}}$ dependence corresponds to the efficiency of probe wave polarization switching. Figure 5f shows the modulation of the probe wave when the polarizations of the two pump tones were switched: the higher-frequency pump wave was moved to the slow axis and the lower-frequency one to the fast axis, and ${{\Omega }}$ set to 2*π* × 169 MHz. In this case, the wavelength of maximum probe modulation $\lambda _{\mathrm{max}}$ equals 1539.2 nm. This wavelength is shorter than that of the pumps, as expected. Lastly, the modulation of the probe wave is non-reciprocal. Figure 5c (red dashed trace) shows also a second measurement in which the probe wave was launched in the $+ {\hat{\boldsymbol z}}$ direction, alongside the two pumps. All other settings are the same as those of the solid blue trace in the same panel. No modulation of the probe wave is observed above the noise level in this case. The cross-polarization coupling of the probe is inhibited by wavenumber mismatch, as discussed above. Residual polarization switching of co-propagating probe waves is at least 20 dB lower than in the counter-propagating direction.

## Discussion

In this work we reported a comprehensive first study of forward SBS processes in PM fibres. Compared with SMF^15,26,27^, forward SBS spectra in PM fibres are more complex and involve larger number of guided acoustic modes. The observed spectra are in very good agreement with numerical modelling. Two types of processes were identified and characterized: intra-modal forward SBS, in which two optical pump components are co-aligned in a common principal axis, and inter-modal forward SBS driven by orthogonally polarized pumps. Intra-modal forward SBS induces XPM of probe tones that are co-polarized with the two pumps, similar to the equivalent process in SMF. Due to the lack of radial symmetry in the PM fibre cross-section, XPM spectra along the slow and fast axes are distinct. The intra-modal process also leads to cross-polarization phase modulation: pump light in one principal axis leads to the modulation of a probe wave of the orthogonal polarization. The acoustic wave couples between two separate intra-modal forward SBS processes along the two axes, in analogy to BDGs of backwards scattering in PM fibres^42–47^.

Inter-polarization forward SBS was realized in a standard, off-the-shelf fibre for the first time. The two orthogonal pump tones induce an electro-strictive driving force^38^. The acoustic waves stimulated in the inter-modal process may be either co-propagating or counter-propagating with respect to the pumps. Inter-modal scattering is characterized by acoustic axial wavenumbers that are 1000 times larger than those of intra-modal forward SBS. Due to this property, inter-modal forward SBS leads to cross-polarization coupling of probe waves that are counter-propagating with respect to the pumps. This coupling is non-reciprocal: probe waves of the same frequencies that co-propagate with the pumps are unaffected. Such coupling may occur at multiple probe wavelength windows for the same pump wavelength, through different acoustic frequencies of the forward SBS spectrum.

In previous studies, inter-modal forward SBS in photonic integrated circuits has been extended to emit–receive configurations: pump tones launch acoustic waves from one physical waveguide, whereas the inter-modal scattering of optical probe waves takes place in a different one^29–33^. Emit–receive processes with two separate waveguides are unaffected by Kerr nonlinearity. Similar separation may be achieved in multi-core PM fibres^57,60^. The present study was carried out for a specific class of PM fibres, known as panda type. The forward SBS spectra in other types of PM fibres would be different. However, the fundamental characteristics of intra-modal and inter-modal scattering would hold. Forward SBS processes driven by short optical pulses may induce timing jitter and fluctuations in phase and intensity of both acoustic and optical waves^61^. However, these effects remain weak for the short lengths, modest power levels and comparatively long pulses relevant to this study.

The fibre under test used in this work was stripped of its protective coating. Bare fibres exhibit stronger forward SBS, with narrower modal linewidths, as little acoustic energy is transmitted outside the fibre^22–25^. The narrow spectral lines help compare model with measurement and distinguish between closely spaced spectral peaks. The use of bare fibres outside the research laboratory is impractical. However, the magnitude and linewidths of forward SBS in commercially available fibres that are coated with a thin polyimide layer are comparable with those of bare fibres^62,63^. Therefore, the effects found in this work may be demonstrated and employed in coated PM fibres as well.

The non-reciprocal cross-polarization coupling of optical probe waves may be considered as a basis for isolator and circulator devices. Optical isolation based on inter-modal forward SBS has been proposed in chalcogenide glass waveguides^64^ and realized in suspended silicon membrane devices^33^. The extent of cross-coupling reached over the 20 m-long fibre under test was modest: on the order of only −43 dB. However, PM fibres of kilometre-scale lengths are employed, e.g., within fibre-optic gyroscopes^65,66^. The process efficiency scales with the fibre length squared and would reach −10 dB over 1 km of PM fibre (with suitable coating as noted above). The effects of optical losses over kilometre length would still be small. On the other hand, the spectral bandwidth of cross-polarization coupling of probe waves would be inversely proportional to the fibre length, due to wavenumber matching considerations. The bandwidth can be extended through careful design of chromatic and polarization-mode dispersion^64^. The relative non-reciprocity of cross-polarization coupling observed between the two directions of probe wave propagation was rather large: at least 20 dB. Strong cross-polarization coupling might also become a disadvantage: PM fibres are designed to prevent crosstalk between their principal axes. Unintended inter-modal forward SBS over long sections could defeat that purpose.

Forward SBS processes in standard fibres are being used in a promising new class of sensors, towards the analysis of media outside the cladding boundaries where light cannot reach^22–25,62,63^. Measurements are also possible outside polyimide coatings^62,63^ and support the analysis of the coating layers themselves^67^. However, these sensors face a fundamental challenge: forward scattering events are difficult to localize. Although several protocols have been established for indirect distributed mapping of forward SBS^23–25^, they are still limited by considerable complexity, poor signal-to-noise ratios, and modest range and spatial resolution. Most importantly, all known protocols retrieve the local forward SBS response through the spatial derivatives of raw data traces, which are accumulated from the input end to the point of interest^23–25^. The derivative analysis is highly susceptible to additive noise.

By contrast, the cross-polarization coupling of a counter-propagating probe wave through inter-modal forward SBS can be localized directly, similar to well established time-domain reflectometry methods. Preliminary measurements reported above clearly distinguish between air and ethanol outside the cladding of one fibre section. Inter-modal scattering opens up exciting new possibilities for distributed opto-mechanical sensing of media outside the cladding and coating, with potentially longer range, higher resolution, better precision, faster acquisition and simpler systems. The introduction of inter-modal forward SBS to standard fibres is particularly significant in this context: specialty nano-structured fibres are less suitable for distributed sensing applications, as they do not easily scale to kilometre lengths.

In conclusion, inter-modal forward SBS processes and the associated opto-mechanical non-reciprocity were thus far restricted to highly specialized platforms. With the introduction of the effect to standard fibre, further research could be open to a much larger community. Ongoing work addresses potential applications in opto-mechanical isolators and circulators, narrowband microwave-photonic filters, forward SBS laser sources and distributed opto-mechanical sensing over off-the-shelf, standard PM fibres.

## Materials and methods

### Parameters used in forward SBS calculations

The acoustic modal profiles and cut-off frequencies are found by numerical solutions of the elastic equation of motion^68^, using a commercial platform:

8 $${{\Omega }}^2{\mathop{u}\limits^{\rightharpoonup}}_m\left( {x,y} \right) + v_{\mathrm{S}}^2\left( {x,y} \right)\nabla ^2{\mathop{u}\limits^{\rightharpoonup}}_m\left( {x,y} \right) + \left[ {v_{\mathrm{L}}^2\left( {x,y} \right) - v_{\mathrm{S}}^2\left( {x,y} \right)} \right]\nabla [ {\nabla \cdot {\mathop{u}\limits^{\rightharpoonup}}_m\left( {x,y} \right)} ] = 0$$

here ${\mathop{u}\limits^{\rightharpoonup}}_m\left( {x,y} \right)$ denotes the normalized transverse profile of material displacement in acoustic mode $m$ (see also Supplementary Information 1) and $v_{\mathrm{L,S}}\left( {x,y} \right)$ are the velocities of dilatational and shear acoustic waves, respectively. The parameters used for the silica cladding were as follows^69^: outer diameter $d$ = 125.5 μm, density $\rho$ = 2200 kg m^−3^, $v_{\mathrm{L}}$ = 5996 m s^−1^ and $v_{\mathrm{S}}$ = 3740 m s^−1^. The parameters of the B_2_O_3_-doped silica strain rods were as follows^70^: $\rho$ = 2080 kg m^−3^, $v_{\mathrm{L}}$ = 4895 m s^−1^ and $v_{\mathrm{S}}$ = 4100 m s^−1^. The radii of the rods are 17.25 μm and their centres are located ±27.5 μm away from the symmetry axis of the fibre, along the $\pm \hat x$ directions (see Fig. 1b). The mode field diameter of the single optical mode for both polarizations was taken as 10.4 μm.

The decay rates of acoustic energy in each of the two media were modelled as: ${{\Gamma }}\left( {{\Omega }} \right) = {{\Gamma }}_0 + {{\Gamma }}_2{{\Omega }}^2$ ^53^. The coefficients ${{\Gamma }}_{0,2}$ in the silica and strain rods were fitted based on the measured forward SBS spectra. For the silica cladding we used: ${{\Gamma }}_0$ = 1.25 × 10^6^ rad Hz and ${{\Gamma }}_2$ = 1.6 × 10^−12^ rad^−1^ Hz^−1^. The values found for the strain rods were: ${{\Gamma }}_0$ = 7.25 × 10^6^ rad Hz and ${{\Gamma }}_2$ = 1.6 × 10^−12^ rad^−1^ Hz^−1^. For each mode $m$, the linewidth is estimated by a weighted sum of dissipation contributions in the silica cladding and strain rods: ${{\Gamma }}_m = {\iint} {{{\Gamma }}\left( {{{\Omega }}_m,x,y} \right)\rho \left( {x,y} \right)| {{\mathop{u}\limits^{\rightharpoonup}}_m\left( {x,y} \right)} |^2{\mathrm{d}}x{\mathrm{d}}y/} {\iint} {\rho \left( {x,y} \right)| {{\mathop{u}\limits^{\rightharpoonup}}_m\left( {x,y} \right)} |^2{\mathrm{d}}x{\mathrm{d}}y}$.

### Experimental estimate of the nonlinear coefficient of inter-modal forward SBS

Let us denote the instantaneous input optical powers of the two pump tones in an inter-polarization forward SBS process as $P_{1,2}\left( t \right) = \bar P_{1,2}[ {1 + \beta _{1,2}{\mathrm{cos}}\left( {f_{1,2}t} \right)} ]$, where $\bar P_{1,2}$ are the average powers of the pump waves, $f_{1,2}$ are low angular radiofrequencies of lock-in signals (see ‘Measurements and numerical calculations’), $t$ stands for time and $\beta _{1,2}$ denote the pre-calibrated modulation depths of the pump waves. The forward SBS interaction leads to an exchange of power between the two pump tones. The instantaneous output power of pump wave 1 is modulated at the sum radiofrequency $f_1 + f_2$, with a modulation depth given by:

9 $$\beta _{1,{\mathrm{out}}} = \frac{1}{2}\beta _1\beta _2\bar P_2\left[ {2{\mathrm{Im}}\left\{ {\gamma \left( {{\Omega }} \right)} \right\}} \right]L$$

here, ${{\Omega }}$ is the frequency offset between the two stimulating pump waves, $L$ denotes the length of the fibre under test and $\gamma \left( {{\Omega }} \right)$ is the nonlinear coefficient of the inter-polarization forward SBS process. The modulation at $f_1 + f_2$ is detected by lock-in amplifier (see ‘Measurements and numerical calculations’) and the voltage magnitude $V_{\mathrm{out}}$ at the amplifier output is noted.

To calibrate the nonlinear coefficient, a pump wave of the same average power passes through an electro-optic amplitude modulator with known $V_\pi$. The modulator is biased at quadrature and driven by a sine wave of radiofrequency $f_1 + f_2$ and variable magnitude $V_{\mathrm{mod}}$. The modulated optical wave is detected by the same photo-receiver used in the forward SBS measurements and analysed by the lock-in amplifier. The modulator voltage $V_{\mathrm{mod}}$ is varied until the lock-in amplifier output $V_{\mathrm{out}}$ reaches the value recorded earlier in the forward SBS experiments. At the particular drive voltage $V_{\mathrm{mod}} = V_{\mathrm{ref}}$, the modulation depth of the detected optical wave matches that of the forward SBS measurements $\beta _{1,{\mathrm{out}}}$:

10 $$\pi \frac{{V_{\mathrm{ref}}}}{{V_\pi }} = \beta _{1,{\mathrm{out}}} = \frac{1}{2}\beta _1\beta _2\bar P_2\left[ {2{\mathrm{Im}}\left\{ {\gamma \left( {{\Omega }} \right)} \right\}} \right]L$$

It is noteworthy that $V_{\mathrm{ref}} \ll V_\pi$. From Eq. (10), we find:

11 $${\mathrm{Im}}\left\{ {\gamma \left( {{\Omega }} \right)} \right\} = \frac{{\pi V_{\mathrm{ref}}}}{{V_\pi \beta _1\beta _2\bar P_2L}}$$

## Supplementary information

Supplementary analysis
