# Supplementary material for: Forward stimulated Brillouin scattering and opto-mechanical non-reciprocity in standard polarization maintaining fibres
Source: Light Sci Appl. 2021 Jun 7;10:119. doi: 10.1038/s41377-021-00557-y (PMC8180518; doi:10.1038/s41377-021-00557-y)
Supplement: Supplementary file 1 — Supplementary analysis [file 41377_2021_557_MOESM1_ESM.docx]

**Supplementary Information: Forward Stimulated Brillouin Scattering and Opto-Mechanical Non-Reciprocity in Standard Polarization Maintaining Fibres**

*Gil Bashan, Hilel Hagai Diamandi, Yosef London, Kavita Sharma, Keren Shemer, Elad Zehavi and Avi Zadok^*^*

*Faculty of Engineering and Institute for Nano-Technology and Advanced Materials, Bar-Ilan University, Ramat-Gan 5290002, Israel*

*^*^*[*Avinoam.Zadok@biu.ac.il*](mailto:Avinoam.Zadok@biu.ac.il)

**1. Guided acoustic modes of polarization maintaining fibres**

Figure S1b shows a schematic cross-section of a single-mode panda-type PM fibre. The fibre includes two cylindrical rods of silica doped with B_2_O_3_ that are embedded in the cladding. The radii of the rods are 17.25 μm, and their centres are located ±27.5 μm away from the cladding symmetry axis in the transverse $\pm\hat{\boldsymbol{x}}$ directions. Thermal expansion of the rods during fibre production induces permanent strain and birefringence between the $\hat{\boldsymbol{x}}$ axis and the orthogonal transverse direction $\hat{\boldsymbol{y}}$. The effective index $n_{s}$ of $\hat{\boldsymbol{x}}$ - polarized light is higher than $n_{f}$ of $\hat{\boldsymbol{y}}$ polarization. The two directions are referred to as the slow and fast principal axes, respectively. The difference between indices is typically in the fourth decimal point.

Throughout this work, the acoustic oscillations considered are predominantly transverse, and we neglect axial displacement. The modal displacement of mode $m$ (in meters), oscillating at acoustic frequency $\Omega$, may be expressed as^1-3^:

| $\boldsymbol{U}_{m}\left( x,y,z,t \right)=\left[ U_{x}\left( x,y \right)\hat{\boldsymbol{x}}+U_{y}\left( x,y \right)\hat{\boldsymbol{y}} \right]\exp\left[ jq_{m}\left( \Omega\right)z-j\Omega t \right]+c.c.=B_{m}\left( \Omega\right)\boldsymbol{u}_{m}\left( x,y \right)\exp\left[ jq_{m}\left( \Omega\right)z-j\Omega t \right]+c.c.$ | (1) |
| --- | --- |

In Eq. (1), $x,y$ are transverse Cartesian coordinates in the $\hat{\boldsymbol{x}}$ and $\hat{\boldsymbol{y}}$ directions, respectively, $z$ denotes axial position along the fibre and $t$ stands for time. $\boldsymbol{u}_{m}$ [m^-1^] represents the transverse profile of guided acoustic mode $m$. It is normalized so that $\left( 1/{\rho_{1}} \right)\iint\rho\left( x,y \right)\left| \boldsymbol{u}_{m}\left( x,y \right) \right|^{2}dxdy=1$. Here $\rho\left( x,y \right)$ is the local density and $\rho_{1}$ is the density of silica. Also in Eq. (1), $B_{m}$ [m^2^] is the modal displacement magnitude which is determined by electrostrictive driving forces (see later), and $q_{m}\left( \Omega\right)$ is the axial acoustic wavenumber which is frequency dependent. For each mode, there is a cut-off frequency $\Omega_{m}$ below which it may not propagate in the axial direction^1-3^. The cut-off frequencies of modes addressed in this work are hundreds of MHz. When $\Omega$ approaches $\Omega_{m}$, the axial wavenumber $q_{m}$ and group velocity ${\partial\Omega}/{\partial q_{m}}$ approach zero^1-3^. By contrast, the axial phase velocity $\Omega/{q_{m}}$ approaches infinity^1-3^.

The modal profiles and cut-off frequencies are found by numerical solutions of the elastic equation of motion^4^, using a commercial platform:

| $\Omega^{2}\boldsymbol{u}_{m}\left( x,y \right)+v_{S}^{2}\left( x,y \right)\nabla^{2}\boldsymbol{u}_{m}\left( x,y \right)+\left[ {v_{L}^{2}\left( x,y \right)-v}_{S}^{2}\left( x,y \right) \right]\nabla\left[ \nabla\cdot\boldsymbol{u}_{m}\left( x,y \right) \right]=0$ | (2) |
| --- | --- |

Here $v_{L,S}\left( x,y \right)$ are the velocities of dilatational and shear acoustic waves, which vary between the silica cladding and the strain rods. Values used in calculations are listed in Methods within the Main Text. The boundary conditions between cladding and rods require the continuity of normal displacement and normal stress. Throughout this work we assume that the fibre is stripped of its coating and kept in air. The corresponding boundary condition is zero stress at the outer edge of the cladding. The PM fibre cross-section supports large number of guided acoustic modes. Unlike single-mode fibres, the transverse profiles of guided acoustic modes in the PM fibre cannot be broken into separate functions of radial and azimuthal coordinates. Figures S1c and S1d show an example of the normalized transverse profiles of material displacement in the $\hat{\boldsymbol{x}}$ and $\hat{\boldsymbol{y}}$ directions for one guided mode, with a cut-off frequency of 175 MHz.


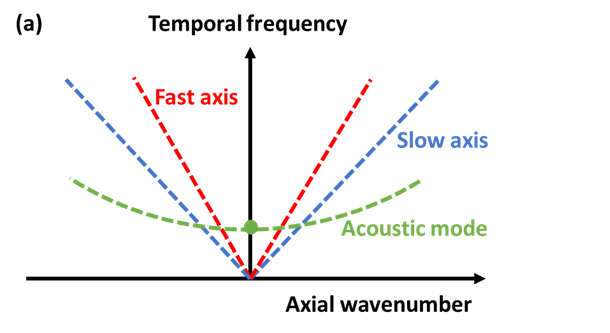

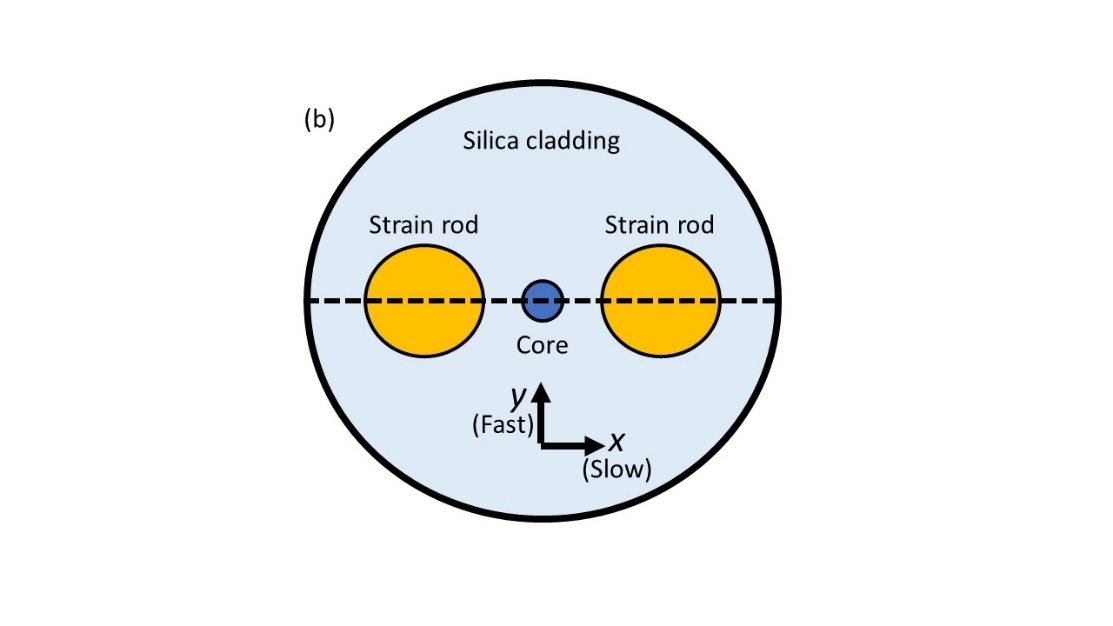


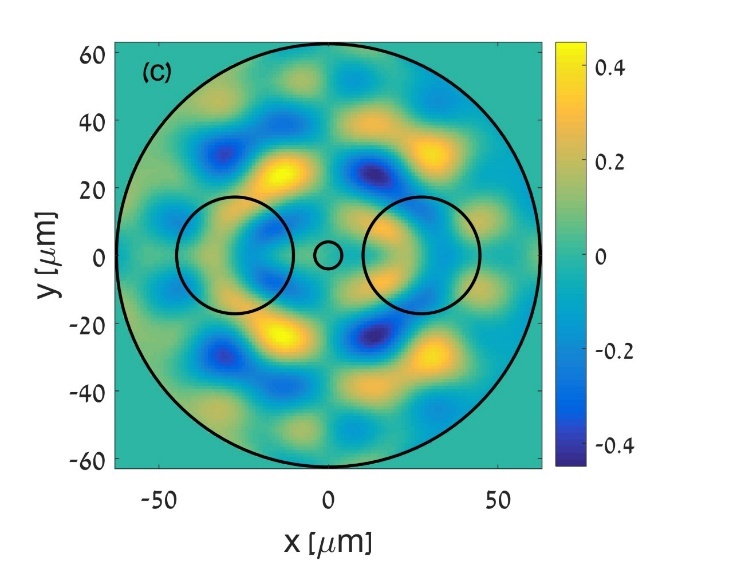

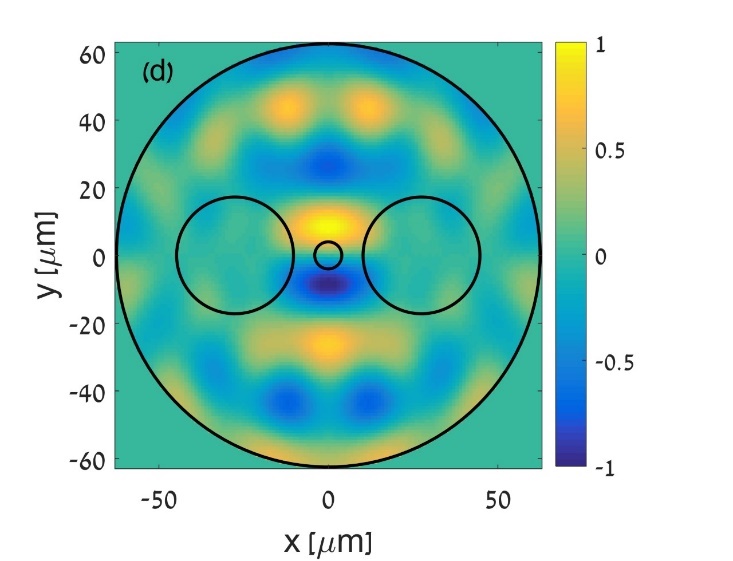


**Supplementary Figure S1.** **Polarization maintaining fibres**. (a) – Schematic illustration of the dispersion relations between temporal frequency and axial wavenumber for light guided in the fast axis (red) and slow axis (blue), and for a guided acoustic mode (green). The circular green marker denotes the modal cut-off frequency. (b) – Schematic cross-section of a panda-type polarization maintaining fibre, with two B_2_O_3_-doped silica strain rods embedded in the pure silica cladding. The centres of the rods are placed at equal distances from the fibre axis in the $\pm\hat{\boldsymbol{x}}$ directions. Thermal expansion of the rods during fibre production induces permanent strain and birefringence in the fibre, with $\hat{\boldsymbol{x}}$ being the slow axis. (c), (d) – Calculated normalized transverse profiles of material displacement in one guided acoustic mode, with a cut-off frequency of 175 MHz. Panels (c) and (d) present displacement components in the $\hat{\boldsymbol{x}}$ and $\hat{\boldsymbol{y}}$ directions, respectively. Black circles denote the boundaries of the cladding, strain rods, and core.

**2. Intra-modal forward stimulated Brillouin scattering in polarization maintaining fibres**

*2.1 Intra-modal stimulation of guided acoustic waves*

Consider two continuous optical pump tones that are polarized along the slow principal axis $\hat{\boldsymbol{x}}$ and co-propagate in the positive $\hat{\boldsymbol{z}}$ direction (Supplementary Figure S2a). Let us denote the optical frequencies of the two tones as $\omega_{p}\pm\frac{1}{2}\Omega$, where $\omega_{p}$ is a central frequency and $\Omega$ represents a radio-frequency detuning variable. The optical power of the two tones combined includes constant terms and a beating pattern. The beating term is propagating with frequency $\Omega$, axial wavenumber $K_{s}={n_{s}\Omega}/c$ and magnitude of $\tilde{P}\left( \Omega\right)=2n\varepsilon_{0}cA_{1}A_{2}^{*}$ [W]. Here $n\approx n_{s,f}$, $\varepsilon_{0}$ is the vacuum permittivity, $c$ represents the speed of light in vacuum, and $A_{1,2}$ are the complex magnitudes [V] of the field components of higher and lower frequencies, respectively. The transverse profile of the single optical mode is noted by $E_{T}\left( r \right)$ [m^-1^], where $r$ is the radial transverse coordinate. It is normalized so that $2\pi\int\left| E_{T}\left( r \right) \right|^{2}rdr=1$. The profile is well approximated by a circular Gaussian shape with a mode-field diameter $2\sqrt{2}w$: $E_{T}\left( r \right)\propto\exp\left[ -{r^{2}}/\left( 2w^{2} \right) \right]$. We disregard small scale differences in $E_{T}\left( r \right)$ between the principal axes. We also assume that the pump tones are undepleted, and neglect for the time being the possible exchange of power between the two along $\hat{\boldsymbol{z}}$.

The electro-strictive force per unit volume $\boldsymbol{F}$ associated with the pump tones consists of transverse components in the radial and azimuthal directions^1-3^:

| $\boldsymbol{F}\left( r,\phi,z,t \right)=\frac{1}{4nc}\boldsymbol{f}\left( r,\phi\right)\tilde{P}\left( \Omega\right)\exp\left( jK_{s}z-j\Omega t \right)+c.c.=\frac{1}{4nc}\left[ f_{r}\left( r,\phi\right)\hat{\boldsymbol{r}}+f_{\phi}\left( r,\phi\right)\hat{\boldsymbol{\phi}} \right]\tilde{P}\left( \Omega\right)\exp\left( jK_{s}z-j\Omega t \right)+c.c.$ | (3) |
| --- | --- |
| $f_{r}\left( r,\phi\right)=-\left[ 2a_{1}+4a_{2}+{2a}_{1}\cos\left( 2\phi\right) \right]\frac{\partial E_{T}\left( r \right)}{\partial r}E_{T}\left( r \right)$ | (4) |
| $f_{\phi}\left( r,\phi\right)=2a_{1}\sin\left( 2\phi\right)\frac{\partial E_{T}\left( r \right)}{\partial r}E_{T}\left( r \right)$ | (5) |

In the above equations $\phi$ is the transverse azimuthal coordinate, and $\hat{\boldsymbol{r}}$ and $\hat{\boldsymbol{\phi}}$ are unit vectors in the radial and azimuthal directions, respectively. The coefficients $a_{1,2}$ are drawn from elements of the photo-elastic tensor $\boldsymbol{p}$ of silica^1-3^: $a_{1}=-n^{4}\left( p_{11}-p_{12} \right)$ = 0.66 and $a_{2}=-n^{4}p_{12}$ = -1.19. If the pump tones are polarized in the fast axis $\hat{\boldsymbol{y}}$ instead, the force $\boldsymbol{F}$ is rotated accordingly, and $K_{s}$ in Eq. (3) is replaced with $K_{f}={n_{f}\Omega}/c$. The transverse dependence of the electro-strictive driving force may be brought to the following form:

| $\boldsymbol{f}=-{\frac{1}{w^{2}}\left\vert E_{T}\left( r \right) \right\vert}^{2}\left[ \left( 2a_{1}+4a_{2} \right)r\hat{\boldsymbol{r}}\boldsymbol{\pm}{2a}_{1}\left( x\hat{\boldsymbol{x}}-y \hat{\boldsymbol{y}} \right) \right]$ | (6) |
| --- | --- |

The force consists of a first term which is radially symmetric and independent of pumps polarization, and a second term that depends on the choice of axis. The ± signs in Eq. (6) correspond to pump waves along the $\hat{\boldsymbol{y}}$ and $\hat{\boldsymbol{x}}$ directions, respectively.

The electrostrictive force serves as a driving term for the elastic wave equation. The force leads to the stimulation of acoustic waves with frequency $\Omega$, axial wavenumber $q_{m}$ of either $K_{s}$ or $K_{f}$, and modal magnitude that is given by^1-3^:

| $B_{m}\left( \Omega\right)=\frac{1}{{4nc\rho}_{1}}\frac{\iint\boldsymbol{u}_{m}^{\dagger}\left( x,y \right)\cdot\boldsymbol{f}\left( x,y \right)dxdy}{\Omega_{m}^{2}-\Omega^{2}-j\Gamma_{m}\Omega}\tilde{P}\left( \Omega\right)=\frac{1}{{4nc\rho}_{1}}Q_{\mathrm{ES}}^{\left( m \right)}H_{m}\left( \Omega\right)\tilde{P}\left( \Omega\right)$ | (7) |
| --- | --- |

The efficiency of stimulation scales with the spatial overlap integral $Q_{\mathrm{ES}}^{\left( m \right)}=\iint\boldsymbol{u}_{m}^{\dagger}\left( x,y \right)\cdot\boldsymbol{f}\left( x,y \right)dxdy$ between the transverse profiles of the electrostrictive driving force and the modal displacement^1-3^. Note that the overlap integrals for $\hat{\boldsymbol{x}}$ polarized or $\hat{\boldsymbol{y}}$ polarized pump waves are not the same, due to changes in $\boldsymbol{f}$ and the lack of radial symmetry in the acoustic modal displacement profiles $\boldsymbol{u}_{m}\left( x,y \right)$. Also in Eq. (7), $\Gamma_{m}$ denotes the modal linewidth, which also signifies the decay rate of acoustic intensity. For bare fibres in air, there is no loss of acoustic energy to the surroundings and the linewidths $\Gamma_{m}$ are determined entirely by internal acoustic dissipation in the silica cladding and strain rods. Dissipation is modelled by second-order dependence on frequency $\Omega$^5^: $\Gamma\left( \Omega\right)=\Gamma_{0}+\Gamma_{2}\Omega^{2}$, in each of the two media. The coefficients $\Gamma_{0,2}$ for silica and strain rods are fitted experimentally and listed in Methods in the Main Text. For each mode $m$, the linewidth is estimated by a weighted sum of contributions in the silica cladding and strain rods: $\Gamma_{m}={\iint\Gamma\left( \Omega_{m},x,y \right){\rho\left( x,y \right)\left| \boldsymbol{u}_{m}\left( x,y \right) \right|}^{\boldsymbol{2}}dxdy}/{\iint{\rho\left( x,y \right)\left| \boldsymbol{u}_{m}\left( x,y \right) \right|}^{\boldsymbol{2}}dxdy}$. For brevity, we represent hereunder the radio-frequency response of the modal acoustic stimulation by $H_{m}\left( \Omega\right)=1/\left( \Omega_{m}^{2}-\Omega^{2}-j\Gamma_{m}\Omega\right)$. The stimulation of acoustic mode $m$ is maximal at its cut-off frequency $\Omega=\Omega_{m}$.

Let us denote the transverse strain tensor associated with the stimulated modal displacement as $\boldsymbol{S}_{m}\left( x,y,z,t \right)=B_{m}\left( \Omega\right)\boldsymbol{s}_{m}\left( x,y \right)\exp\left( jK_{s,f}z-j\Omega t \right)+c.c$, with: $s_{m,xx}={\partial u_{m,x}}/{\partial x}$, $s_{m,yy}={\partial u_{m,y}}/{\partial y}$ and $s_{m,xy}= s_{m,yx}=\frac{1}{2}\left( {\partial u_{m,x}}/{\partial y}+{\partial u_{m,y}}/{\partial x} \right)$. Here $u_{m,x}$ and $u_{m,y}$ denote the components of the normalized modal displacement profile $\boldsymbol{u}_{m}\left( x,y \right)$ in the $\hat{\boldsymbol{x}}$ and $\hat{\boldsymbol{y}}$ directions, respectively. Strain gives rise to photo-elastic perturbations $\boldsymbol{\delta}\boldsymbol{\varepsilon}_{m}\left( x,y,z,t \right)$ in the dielectric tensor $\boldsymbol{\varepsilon}$ ^1-3,6^:

| $\left( \begin{matrix} \delta\varepsilon_{m,xx} \\ \delta\varepsilon_{m,yy} \\ \delta\varepsilon_{m,xy} \end{matrix} \right)=-\frac{1}{4nc\rho_{1}}Q_{\mathrm{ES}}^{\left( m \right)}H_{m}\left( \Omega\right)n^{4}\left( \begin{matrix} p_{11} & p_{12} & 0 \\ p_{12} & p_{11} & 0 \\ 0 & 0 & p_{44} \end{matrix} \right)\left( \begin{matrix} s_{m,xx} \\ s_{m,yy} \\ {2s}_{m,xy} \end{matrix} \right)\tilde{P}\left( \Omega\right)\exp\left( jK_{s,f}z-j\Omega t \right)+c.c$  $=\frac{1}{{4nc\rho}_{1}}Q_{\mathrm{ES}}^{\left( m \right)}H_{m}\left( \Omega\right)\left( \begin{matrix} \mu_{m,xx} \\ \mu_{m,yy} \\ \mu_{m,xy} \end{matrix} \right)\tilde{P}\left( \Omega\right)\exp\left( jK_{s,f}z-j\Omega t \right)+c.c.$ | (8) |
| --- | --- |

Here $p_{44}=\frac{1}{2}\left( p_{11}-p_{12} \right)$, and the elements of the tensor $\boldsymbol{\mu}_{m}\left( x,y \right)$ are given by:

| $\left( \begin{matrix} \mu_{m,xx} \\ \mu_{m,yy} \\ \mu_{m,xy} \end{matrix} \right)=-n^{4}\left( \begin{matrix} p_{11} & p_{12} & 0 \\ p_{12} & p_{11} & 0 \\ 0 & 0 & p_{44} \end{matrix} \right)\left( \begin{matrix} s_{m,xx} \\ s_{m,yy} \\ {2s}_{m,xy} \end{matrix} \right)$ | (9) |
| --- | --- |

The effective dielectric perturbation seen by the optical mode is given by^1-3^:

| $\bar{\delta\varepsilon}_{m,ij}\left( z,t \right)=\iint\delta\varepsilon_{m,ij}\left( x,y,z,t \right)\left\vert E_{T}\left( x,y \right) \right\vert^{2}dxdy=\frac{1}{{4nc\rho}_{1}}Q_{\mathrm{ES}}^{\left( m \right)}H_{m}\left( \Omega\right)\left[ \iint\mu_{m,ij}\left( x,y \right)\left\vert E_{T}\left( x,y \right) \right\vert^{2}dxdy \right]\tilde{P}\left( \Omega\right)\exp\left( jK_{s,f}z-j\Omega t \right)+c.c=\frac{1}{{4nc\rho}_{1}}Q_{\mathrm{ES}}^{\left( m \right)}Q_{\mathrm{PE},ij}^{\left( m \right)}H_{m}\left( \Omega\right)\tilde{P}\left( \Omega\right)\exp\left( jK_{s,f}z-j\Omega t \right)+c.c.$ | (10) |
| --- | --- |

Here $i,j=x,y$ and $Q_{\mathrm{PE},ij}^{\left( m \right)}=\iint\mu_{m,ij}\left( x,y \right)\left| E_{T}\left( x,y \right) \right|^{2}dxdy$ denote transverse spatial overlap integrals between elements of the photo-elastic perturbation to the dielectric tensor and the optical mode. In standard single-mode fibres, the perturbation tensors ${\bar{\boldsymbol{\delta\varepsilon}}}_{m}\left( z,t \right)$ for purely radial acoustic modes reduce to scalar values which signify refractive index modulation that is polarization-independent^1-3^.

*2.2 Scattering of probe waves by intra-modal forward stimulated Brillouin scattering processes*

Consider next a continuous optical probe of frequency $\omega_{sig}$ that is co-propagating with the pump tones and polarized along the slow axis $\hat{\boldsymbol{x}}$, with wavenumber $k_{sig,s}={n_{s}\omega_{\mathrm{sig}}}/c$ and magnitude $A_{\mathrm{sig}}$. A stimulated acoustic mode gives rise to nonlinear polarization terms at frequencies $\omega_{\mathrm{sig}}\pm\Omega$. When the two pump tones are also polarized along the $\hat{\boldsymbol{x}}$ axis, these terms are of the following forms^7^:

| $\boldsymbol{P}_{m,+\Omega}^{\mathrm{NL}}\left( x,y,z,t \right)=\varepsilon_{0}A_{\mathrm{sig}}E_{T}\left( x,y \right)\frac{1}{4nc\rho_{1}}Q_{\mathrm{ES}}^{\left( m \right)}H_{m}\left( \Omega\right)\tilde{P}\left( \Omega\right)\exp\left[ j\left( {k_{sig,s}+K}_{s} \right)z-j\left( \omega_{\mathrm{sig}}+\Omega\right)t \right]\boldsymbol{\mu}_{m}\left( x,y \right)\left( \begin{matrix} 1 \\ 0 \end{matrix} \right)+c.c.$ | (11) |
| --- | --- |
| $\boldsymbol{P}_{m,-\Omega}^{\mathrm{NL}}\left( x,y,z,t \right)=\varepsilon_{0}A_{\mathrm{sig}}E_{T}\left( x,y \right)\frac{1}{4nc\rho_{1}}\left[ Q_{\mathrm{ES}}^{\left( m \right)}H_{m}\left( \Omega\right)\tilde{P}\left( \Omega\right) \right]^{*}\exp\left[ j\left( {k_{sig,s}-K}_{s} \right)z-j\left( \omega_{\mathrm{sig}}-\Omega\right)t \right]\boldsymbol{\mu}_{m}^{\dagger}\left( x,y \right)\left( \begin{matrix} 1 \\ 0 \end{matrix} \right)+c.c.$ | (12) |

The two terms may drive the scattering of the incident probe wave into sidebands of frequencies $\omega_{\mathrm{sig}}\pm\Omega$. The complex magnitudes of the sideband fields along the slow axis, $A_{x\pm}\left( z \right)$, propagate with wavenumbers ${k_{sig,s}\pm K}_{s}$. The sidebands $A_{y\pm}\left( z \right)$ at the orthogonal polarization, if generated, would take up different wavenumbers due to the PM fibre birefringence: ${k_{sig,f}\pm K}_{f}$ where $k_{sig,f}={n_{f}\omega_{\mathrm{sig}}}/c$. We assume hereunder that the generated sidebands are sufficiently weak so that the magnitude of the input probe wave $A_{\mathrm{sig}}$ at $\omega_{\mathrm{sig}}$ remains constant. The sidebands build-up is governed by a pair of nonlinear vector wave equations. Subject to the slowly varying envelope approximations, these take up the forms:

| $2jk_{\mathrm{sig}}E_{T}\left( x,y \right)\frac{d}{dz}\left( \begin{matrix} A_{x+}\left( z \right) \\ A_{y+}\left( z \right) \end{matrix} \right)=-\frac{\omega_{\mathrm{sig}}^{2}}{c^{2}}A_{\mathrm{sig}}E_{T}\left( x,y \right)\frac{1}{{4nc\rho}_{1}}Q_{\mathrm{ES}}^{\left( m \right)}H_{m}\left( \Omega\right)\tilde{P}\left( \Omega\right)\left( \begin{matrix} \mu_{m,xx} \\ \mu_{m,yx}\exp\left( j\Delta k_{sig,sf}z \right) \end{matrix} \right)$ | (13) |
| --- | --- |
| $2jk_{\mathrm{sig}}E_{T}\left( x,y \right)\frac{d}{dz}\left( \begin{matrix} A_{x-}\left( z \right) \\ A_{y-}\left( z \right) \end{matrix} \right)=-\frac{\omega_{\mathrm{sig}}^{2}}{c^{2}}A_{\mathrm{sig}}E_{T}\left( x,y \right)\frac{1}{{4nc\rho}_{1}}\left[ Q_{\mathrm{ES}}^{\left( m \right)}H_{m}\left( \Omega\right)\tilde{P}\left( \Omega\right) \right]^{*}\left( \begin{matrix} \mu_{m,xx}^{*} \\ \mu_{m,xy}^{*}\exp\left( j\Delta k_{sig,sf}z \right) \end{matrix} \right)$ | (14) |

Here we approximate $\omega_{\mathrm{sig}}\pm\Omega\approx\omega_{\mathrm{sig}}$ and $\left( {k_{sig,s}\pm K}_{s} \right)\approx\left( k_{sig,f}\pm K_{f} \right)\approx k_{\mathrm{sig}}$. The evolution of sidebands in the input state of polarization $\hat{\boldsymbol{x}}$ is inherently wavenumber matched. By contrast, the potential build-up of sidebands in the orthogonal principal axis is subject to a wavenumber mismatch: $\left( {k_{sig,s}\pm K}_{s} \right)-\left( k_{sig,f}\pm K_{f} \right)\approx k_{sig,s}-k_{sig,f}=\omega_{\mathrm{sig}}\left( n_{s}-n_{f} \right)/c\equiv\Delta k_{sig,sf}$. This mismatch in common PM fibres is on the order of 1,000 rad×m^-1^. Hence, we find that the acoustic waves driven by intra-modal forward SBS cannot give rise to cross-polarization coupling of light: They lack the necessary axial wavenumber to compensate for the fibre birefringence^7^. In the following, therefore, we consider the generation of $\hat{\boldsymbol{x}}$ polarized sidebands only. Multiplying both sides of the nonlinear wave equations by $E_{T}^{*}\left( x,y \right)$ and integrating over the transverse cross-section yields:

| $\frac{d}{dz}A_{x+}\left( z \right)=j\frac{k_{0}}{2n}\frac{1}{{4nc\rho}_{1}}Q_{\mathrm{ES}}^{\left( m \right)}{Q_{\mathrm{PE},xx}^{\left( m \right)}H}_{m}\left( \Omega\right)\tilde{P}\left( \Omega\right)A_{\mathrm{sig}}$ | (15) |
| --- | --- |
| $\frac{d}{dz}A_{x-}\left( z \right)=j\frac{k_{0}}{2n}\frac{1}{{4nc\rho}_{1}}\left[ Q_{\mathrm{ES}}^{\left( m \right)}Q_{\mathrm{PE},xx}^{\left( m \right)}H_{m}\left( \Omega\right)\tilde{P}\left( \Omega\right) \right]^{*}A_{\mathrm{sig}}$ | (16) |

Here $k_{0}={k_{\mathrm{sig}}}/n$ is the vacuum wavenumber. We may now define an equivalent nonlinear coefficient for the intra-modal forward SBS process, in units of W^-1^× m^-1^:

| $\gamma_{m}\left( \Omega\right)=\frac{{k_{0}Q}_{\mathrm{ES}}^{\left( m \right)}Q_{\mathrm{PE},xx}^{\left( m \right)}}{8n^{2}{c\rho}_{1}}H_{m}\left( \Omega\right)$ | (17) |
| --- | --- |

The forward SBS coefficient assumes its largest magnitude at the modal cut-off frequency:

| $\gamma_{0m}=\gamma_{m}\left( \Omega_{m} \right)=j\frac{{k_{0}Q}_{\mathrm{ES}}^{\left( m \right)}Q_{\mathrm{PE},xx}^{\left( m \right)}}{8n^{2}{c\rho}_{1}\Gamma_{m}\Omega_{m}}$ | (18) |
| --- | --- |

The largest values of $\gamma_{0m}$ in bare panda-type PM fibres are on the order of 3-5 W^-1^× m^-1^.

Using the above definition, the nonlinear wave equations for the two sidebands become:

| $\frac{d}{dz}A_{x+}\left( z \right)=j\gamma_{m}\left( \Omega\right)\tilde{P}\left( \Omega\right)A_{\mathrm{sig}}$ | (19) |
| --- | --- |
| $\frac{d}{dz}A_{x-}\left( z \right)=j\gamma_{m}^{*}\left( \Omega\right)\left[ \tilde{P}\left( \Omega\right) \right]^{*}A_{\mathrm{sig}}$ | (20) |

The squared magnitude of the two sidebands are equal: $\left| A_{x\pm}\left( z \right) \right|^{2}=\left| \gamma_{m}\left( \Omega\right) \right|^{2}\left| \tilde{P}\left( \Omega\right) \right|^{2}z^{2}\left| A_{\mathrm{sig}} \right|^{2}$. Their phases, however, are different, and given by: $\pi/2+\arg\left( A_{\mathrm{sig}} \right)\pm\left\{ \arg\left[ \gamma_{m}\left( \Omega\right) \right]+\arg\left[ \tilde{P}\left( \Omega\right) \right] \right\}$. The beating of the two sidebands with the input probe carrier at $\omega_{\mathrm{sig}}$ cancels out and does not represent intensity modulation of the probe wave at frequency $\Omega$. Intra-modal forward SBS along the slow axis therefore contributes cross-phase modulation (XPM) between co-polarized pump and probe waves of different frequencies (Supplementary Figure S2a). This effect is analogous to forward SBS in SMFs, which was characterized extensively^1-3.5^. The above analysis has been restricted to small-signal, weak modulation of the probe wave. When the pump waves are strong enough and/or the fibre under test is long enough, the scattering of the probe wave would lead to the formation of additional, higher-order sidebands^8-9^. However, even in that more general case, the effect on the probe wave is that of phase modulation^8-9^. The complete description of stronger probe wave modulation is outside the scope of this discussion and has been provided elsewhere^8-9^.

We consider next the scenario in which the input probe wave remains polarized along the slow axis $\hat{\boldsymbol{x}}$**,** however intra-modal forward SBS process is driven by two pump tones in the fast axis $\hat{\boldsymbol{y}}$. The wavenumber of the stimulated acoustic wave in this case equals $K_{f}$, rather than $K_{s}$. This change contributes additional wavenumber mismatch in the nonlinear wave equations: $\mp\left( K_{s}-K_{f} \right)=\mp\Omega\left( n_{s}-n_{f} \right)/c\equiv\mp\Delta K_{\mathrm{sf}}$ for the upper and lower sidebands, respectively. $\Delta K_{\mathrm{sf}}$ is smaller than the wavenumber mismatch $\Delta k_{sig,sf}$ between optical frequencies by six orders of magnitude: It is on the order of 0.001 rad×m^-1^. The fibres used in this work are only few meters long, hence $\Delta K_{\mathrm{sf}}$ has little effect on the propagation of the probe wave, and the analysis leading to Eq. (19) and (20) remains valid. Therefore, the intra-modal forward SBS process driven by $\hat{\boldsymbol{y}}$ polarized pumps still gives rise to inter-polarization XPM of probe light in the $\hat{\boldsymbol{x}}$ axis (Supplementary Figure S2b). Note again that the nonlinear coefficients $\gamma_{m}\left( \Omega\right)$ for the inter-polarization and intra-polarization XPM contributions are not the same: The transverse dependence of the electro-strictive forces $\boldsymbol{f}$ and thus the overlap integrals $Q_{\mathrm{ES}}^{\left( m \right)}$ are different.


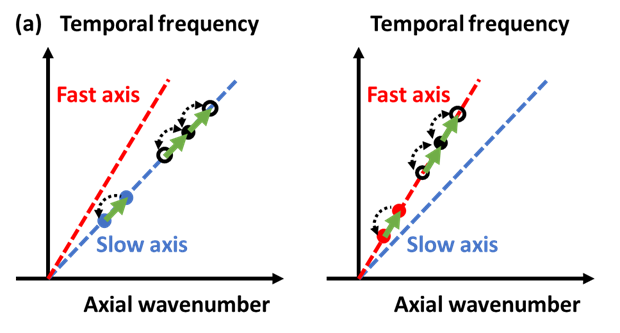

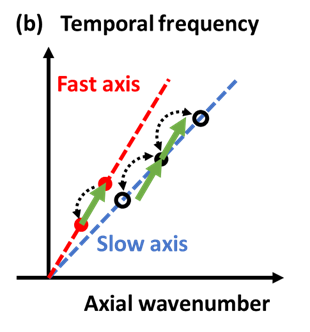


**Supplementary Figure S2.** **Intra-polarization** **forward stimulated Brillouin scattering processes in polarization maintaining fibres**. (a) – Schematic illustration of intra-modal forward SBS along the slow axis (left) or fast axis (right). Two pump tones (full blue circular markers for the slow axis process on the left, full red markers for the fast axis process on the right) stimulate a guided acoustic wave (green arrow). The same acoustic wave modulates a co-polarized, co-propagating probe wave (full black circle), and generates two sidebands (empty circular markers). Dashed black arrows denote acoustically induced coupling. (b) - Cross-polarization phase modulation through intra-modal forward SBS. Two pump tones in the fast axis generate an acoustic wave. The acoustic wave, in turn, generates two sidebands of a probe wave polarized along the slow axis. The process is associated with a nonzero wavenumber mismatch, due to the PM fibre birefringence. However, the mismatch is small and has negligible effect over meters-long fibres. An equivalent process may take place with the pump tones along the slow axis and a probe wave aligned with the fast axis instead.

When the pumps are $\hat{\boldsymbol{y}}$ polarized and the probe wavs is $\hat{\boldsymbol{x}}$ polarized, the nonlinear wave equations for the possible generation of a probe wave sidebands along the $\hat{\boldsymbol{y}}$ axis still exhibit large wavenumber mismatches similar to those of Eq. (13) and (14): $\Delta k_{sig,sf}\mp\Delta K_{\mathrm{sf}}\approx\Delta k_{sig,sf}$. Therefore, intra-modal forward SBS cannot lead to cross-polarization coupling in the PM fibre. This restriction holds true when the pumps and probe are either co-polarized or cross-polarized. Finally, the entire treatment of this section would be equally valid if the probe light is polarized along the fast axis $\hat{\boldsymbol{y}}$ instead. Intra-modal forward SBS process driven by pump tones at either the fast or slow axes would lead to XPM of the probe wave as above. In this case, the photo-elastic overlap integral $Q_{\mathrm{PE},yy}^{\left( m \right)}$ would appear in all expressions instead of $Q_{\mathrm{PE},xx}^{\left( m \right)}$. Here too, intra-polarization and inter-polarization XPM spectra would differ.

To conclude this section, standard PM fibres support intra-modal F-SBS processes that are different for pump tones in the slow or fast axes. The processes induce XPM of co-propagating probe waves, that are either co-polarized with the pumps or aligned with the orthogonal polarization. Intra-modal forward SBS cannot lead to cross-polarization coupling of probe waves.

**3. Inter-modal forward stimulated Brillouin scattering in polarization maintaining fibres**

*3.1 Inter-modal stimulation of guided acoustic waves*

We now address the stimulation of guided acoustic modes by two continuous pump tones of orthogonal polarization (Supplementary Figure S3a). A first component of frequency $\omega_{p}+\frac{1}{2}\Omega$, slow axis $\hat{\boldsymbol{x}}$ polarization, wavenumber $k_{p,s}={n_{s}\left( \omega_{0}+\frac{1}{2}\Omega\right)}/c$ and magnitude $A_{1x}\left( z \right)$, and a second at frequency $\omega_{p}-\frac{1}{2}\Omega$, fast axis $\hat{\boldsymbol{y}}$ polarization, wavenumber ${k_{p,f}=n_{f}\left( \omega_{p}-\frac{1}{2}\Omega\right)}/c$ and magnitude $A_{2y}\left( z \right)$. (Note that the definitions of $k_{p,s}$ and $k_{p,f}$ here are different from those of $k_{sig,s}$ and $k_{sig,f}$ in Section 2 earlier). The two orthogonal pump waves induce an electro-strictive driving force that is oscillating at $\Omega$, of the following form^10^:

| $\boldsymbol{F}\left( x,y,z,t \right)=\frac{1}{4nc}\boldsymbol{f}\left( x,y \right)\tilde{P}\left( \Omega,z \right)\exp\left[ j\left( {\Delta k}_{p,sf}+\bar{K}_{\mathrm{sf}} \right)z-j\Omega t \right]+c.c.=\frac{1}{4nc}{2a}_{1}\left[ E_{T}\left( x,y \right)\frac{{\partial E}_{T}\left( x,y \right)}{\partial y}\hat{\boldsymbol{x}}+E_{T}\left( x,y \right)\frac{{\partial E}_{T}\left( x,y \right)}{\partial x}\hat{\boldsymbol{y}} \right]\tilde{P}\left( \Omega,z \right)\exp\left[ j\left( {\Delta k}_{p,sf}+\bar{K}_{\mathrm{sf}} \right)z-j\Omega t \right]+c.c.$ | (21) |
| --- | --- |

Here we denote once again $\tilde{P}\left( \Omega,z \right)=2n\varepsilon_{0}cA_{1x}\left( z \right)A_{2y}^{*}\left( z \right)$ as before. The axial wavenumber terms of the driving force are ${\Delta k}_{p,sf}=\omega_{p}\left( n_{s}-n_{f} \right)/c$ and $\bar{K}_{\mathrm{sf}}=\frac{1}{2}\Omega\left( n_{s}+n_{f} \right)/c$. Carrying out the transverse derivatives in Eq. (21) yields the following transverse profile for the electrostrictive driving force:

| $\boldsymbol{f}=\frac{2a_{1}}{w^{2}}\left\vert E_{T}\left( x,y \right) \right\vert^{2}\left( y\hat{\boldsymbol{x}}+x\hat{\boldsymbol{y}} \right)=\frac{{2a}_{1}}{w^{2}}\left\vert E_{T}\left( x,y \right) \right\vert^{2}\left( b_{45}{\hat{\boldsymbol{e}}}_{45}\boldsymbol{-}b_{-45}{\hat{\boldsymbol{e}}}_{-45} \right)$ | (22) |
| --- | --- |

Here ${\hat{\boldsymbol{e}}}_{\pm45}\boldsymbol{=}\left( 1/\sqrt{2} \right)\left( \hat{\boldsymbol{x}}\pm\hat{\boldsymbol{y}} \right)$ are unit Jones vectors of linear polarizations oriented at ±45 degrees with respect to the principal axes, and the projections $b_{\pm45}$ are given by $\left( 1/\sqrt{2} \right)\left( x\pm y \right)$. The mode field diameter is noted by $2\sqrt{2}w$ as before. Compared with intra-modal forward SBS, the inter-modal driving force does not include a radially symmetric term and its remaining component is rotated by 45 degrees. The driving force may stimulate the oscillations of guided acoustic modes of frequency $\Omega$, with the following modal magnitude (see Eq. (7) above, repeated here for convenience):

| $B_{m}\left( \Omega,z \right)=\frac{1}{{4nc\rho}_{1}}\frac{\iint\boldsymbol{u}_{m}^{\dagger}\left( x,y \right)\cdot\boldsymbol{f}\left( x,y \right)dxdy}{\Omega_{m}^{2}-\Omega^{2}-j\Gamma_{m}\Omega}\tilde{P}\left( \Omega,z \right)=\frac{1}{{4nc\rho}_{1}}Q_{\mathrm{ES}}^{\left( m \right)}H_{m}\left( \Omega\right)\tilde{P}\left( \Omega,z \right)$ | (23) |
| --- | --- |

This time, however, the acoustic modes are driven by two pump waves of orthogonal polarizations, through an inter-modal process. This property manifests in a much larger axial wavenumber of the acoustic wave: $q_{m}=\left( {\Delta k}_{p,sf}+\bar{K}_{\mathrm{sf}} \right)\approx{\Delta k}_{p,sf}$, on the order of 1,000 rad×m^-1^. This wavenumber is in marked contrast with those of the acoustic waves that are stimulated via intra-modal forward SBS: $q_{m}=K_{s,f}=n_{s,f}\Omega/c$, on the order of 1 rad×m^-1^. Even in the inter-modal case, $q_{m}$ remains much smaller than $\left| \vec{q_{L}} \right|=\Omega/{V_{L}}$ or $\left| \vec{q_{S}} \right|=\Omega/{V_{S}}$ in the silica cladding or the strain rods. Therefore, the acoustic waves are still predominantly transverse. Nevertheless, the axial wavenumber component is non-negligible and may lead to non-reciprocal polarization switching of probe waves, as discussed later. The electro-strictive transverse overlap integral $Q_{\mathrm{ES}}^{\left( m \right)}$ is different from those of the intra-modal forward SBS processes, since the term $\boldsymbol{f}$ is not the same.

In the analysis above, the optical pump tone of the higher frequency $\omega_{p}+\frac{1}{2}\Omega$ was polarized along the slow axis, where the wavenumber is larger. As seen in Eq. (21), the stimulated acoustic wave in that case would be co-propagating with the two pump tones. However, inter-modal forward SBS could stimulate counter-propagating acoustic waves as well. Suppose that the higher-frequency pump tone is polarized along the fast $\hat{\boldsymbol{y}}$ axis instead, with a wavenumber ${n_{f}\left( \omega_{p}+\frac{1}{2}\Omega\right)}/c$, whereas the lower-frequency pump wave is polarized in the slow $\hat{\boldsymbol{x}}$ axis with wavenumber ${n_{s}\left( \omega_{p}-\frac{1}{2}\Omega\right)}/c$. In that case, the electrostrictive driving force at frequency $\Omega$ takes up an axial wavenumber $q_{m}=\left( {-\Delta k}_{p,sf}+\bar{K}_{\mathrm{sf}} \right)\approx{-\Delta k}_{p,sf}<0$. Consequently, the stimulated acoustic waves would be counter-propagating with respect to the two pumps (Supplementary Figure S3a, right).

*3.2 Stimulated amplification in inter-modal forward stimulated Brillouin scattering in polarization maintaining fibres*

We examine next the counter-effect of the stimulated acoustic waves on the magnitudes of the two pump tones $A_{1x,2y}\left( z \right)$. We consider the case in which the high-frequency pump wave is polarized along the slow $\hat{\boldsymbol{x}}$ axis, the lower-frequency pump in the fast $\hat{\boldsymbol{y}}$ axis, and the stimulated acoustic waves is co-propagating with the two pumps as in Eq. (21) (Supplementary Figure S3a, left). The nonlinear polarization term at the upper frequency $\omega_{p}+\frac{1}{2}\Omega$ is given by the following vector:

| $\boldsymbol{P}_{m,+\frac{\Omega}{2}}^{\mathrm{NL}}\left( x,y,z,t \right)=\varepsilon_{0}A_{2y}\left( z \right)E_{T}\left( x,y \right)\frac{1}{{4nc\rho}_{1}}Q_{\mathrm{ES}}^{\left( m \right)}H_{m}\left( \Omega\right)\tilde{P}\left( \Omega,z \right)\exp\left[ j\left( k_{p,f}+{\Delta k}_{p,sf}+\bar{K}_{\mathrm{sf}} \right)z-j\left( \omega_{p}+\frac{1}{2}\Omega\right)t \right]\boldsymbol{\mu}_{m}\left( x,y \right)\left( \begin{matrix} 0 \\ 1 \end{matrix} \right)+c.c.$ | (24) |
| --- | --- |

Note that $\left( k_{p,f}+{\Delta k}_{p,sf}+\bar{K}_{\mathrm{sf}} \right)=k_{p,s}$. The nonlinear polarization vector may affect the optical pump of frequency $\omega_{p}+\frac{1}{2}\Omega$ along the slow axis $A_{1x}\left( z \right)$. In principle, it might also generate an optical wave at the same frequency along the fast axis: $A_{1y}\left( z \right)$. We show below, however, that wavenumber mismatch considerations inhibit this process.

Similarly, the nonlinear polarization vector for the lower frequency $\omega_{p}-\frac{1}{2}\Omega$ is given by:

| $\boldsymbol{P}_{m,-\frac{\Omega}{2}}^{\mathrm{NL}}\left( x,y,z,t \right)=\varepsilon_{0}A_{1x}\left( z \right)E_{T}\left( x,y \right)\frac{1}{{4nc\rho}_{1}}\left[ Q_{\mathrm{ES}}^{\left( m \right)}H_{m}\left( \Omega\right)\tilde{P}\left( \Omega,z \right) \right]^{*}\exp\left[ j\left( k_{p,s}-{\Delta k}_{p,sf}-\bar{K}_{\mathrm{sf}} \right)z-j\left( \omega_{p}-\frac{1}{2}\Omega\right)t \right]\boldsymbol{\mu}_{m}^{\dagger}\left( x,y \right)\left( \begin{matrix} 1 \\ 0 \end{matrix} \right)+c.c.$ | (25) |
| --- | --- |

Here $\left( k_{p,s}-{\Delta k}_{p,sf}-\bar{K}_{\mathrm{sf}} \right)=k_{p,f}$. This nonlinear polarization component is linked with the propagation of the lower-frequency pump wave along the fast axis $A_{2y}\left( z \right)$, and the potential generation of a component at $\omega_{p}-\frac{1}{2}\Omega$ frequency along the slow axis, $A_{2x}\left( z \right)$.

We substitute the nonlinear polarization terms in a pair of nonlinear wave equations, as in Eq. (13) and (14). After multiplying both sides by $E_{T}^{*}\left( x,y \right)$ as before, and integrating over the transverse cross-sections, the equations take up the following forms:

| $2jk_{p}\frac{d}{dz}\left( \begin{matrix} A_{1x}\left( z \right) \\ A_{1y}\left( z \right) \end{matrix} \right)=-\frac{\omega_{p}^{2}}{c^{2}}A_{2y}\left( z \right)\frac{1}{{4nc\rho}_{1}}Q_{\mathrm{ES}}^{\left( m \right)}H_{m}\left( \Omega\right)\tilde{P}\left( \Omega,z \right)\left( \begin{matrix} Q_{\mathrm{PE},xy}^{\left( m \right)} \\ Q_{\mathrm{PE},yy}^{\left( m \right)}\exp\left( j\Delta k_{p,sf}z-jK_{f}z \right) \end{matrix} \right)$ | (26) |
| --- | --- |
| $2jk_{p}\frac{d}{dz}\left( \begin{matrix} A_{2x}\left( z \right) \\ A_{2y}\left( z \right) \end{matrix} \right)=-\frac{\omega_{p}^{2}}{c^{2}}A_{1x}\left( z \right)\frac{1}{{4nc\rho}_{1}}\left[ Q_{\mathrm{ES}}^{\left( m \right)}H_{m}\left( \Omega\right)\tilde{P}\left( \Omega,z \right) \right]^{*}\left( \begin{matrix} Q_{\mathrm{PE},xx}^{\left( m \right)*}\exp\left( -j\Delta k_{p,sf}z+jK_{s}z \right) \\ Q_{\mathrm{PE},yx}^{\left( m \right)*} \end{matrix} \right)$ | (27) |

Here we approximate $\omega_{p}\pm\frac{1}{2}\Omega\approx\omega_{p}$ and $k_{p,s}\approx k_{p,f}\approx k_{p}=n{\omega_{p}}/c$. The equations suggest that the inter-modal forward SBS process may couple between the two pump tones: $A_{1x}\left( z \right)$ and $A_{2y}\left( z \right)$. On the other hand, the potential generation of intra-polarization sidebands, $A_{1y}\left( z \right)$ or $A_{2x}\left( z \right)$, is restricted by considerable wavenumber mismatch: $\left( \Delta k_{p,sf}-K_{f} \right)\approx\Delta k_{p,sf}$ and $\left( -\Delta k_{p,sf}+K_{s} \right)\approx-\Delta k_{p,sf}$, on the order of ±1,000 rad×m^-1^. The coupling terms between the two input pump tones are therefore the only ones remaining. The pair of equations may be written as:

| $\frac{d}{dz}A_{1x}\left( z \right)=j\gamma_{m}\left( \Omega\right)\tilde{P}\left( \Omega,z \right)A_{2y}\left( z \right)$ | (28) |
| --- | --- |
| $\frac{d}{dz}A_{2y}\left( z \right)=j\gamma_{m}^{*}\left( \Omega\right)\left[ \tilde{P}\left( \Omega,z \right) \right]^{*}A_{1x}\left( z \right)$ | (29) |

With a suitable nonlinear coefficient:

| $\gamma_{m}\left( \Omega\right)=\frac{{k_{0}Q}_{\mathrm{ES}}^{\left( m \right)}Q_{\mathrm{PE},xy}^{\left( m \right)}}{8n^{2}c\rho_{1}}H_{m}\left( \Omega\right)$ | (30) |
| --- | --- |

As before, $k_{0}={k_{p}}/n$ represents the vacuum wavenumber. Note that due to the symmetry of the strain tensor, $Q_{\mathrm{PE},xy}^{\left( m \right)}=Q_{\mathrm{PE},yx}^{\left( m \right)}$. The largest magnitude values of $\gamma_{m}\left( \Omega\right)$ in bare panda-type PM fibres are on the order of 1 W^-1^× m^-1^.

The pair of equations may be converted to describe the propagation of the optical powers of the two tones, $P_{1,2}\left( z \right)={2nc\varepsilon_{0}\left| A_{1x,2y}\left( z \right) \right|}^{2}$:

| $\frac{d}{dz}P_{1}\left( z \right)=-2\mathrm{Im}\left\{ \gamma_{m}\left( \Omega\right) \right\}P_{1}\left( z \right)P_{2}\left( z \right)$ | (31) |
| --- | --- |
| $\frac{d}{dz}P_{2}\left( z \right)=2\mathrm{Im}\left\{ \gamma_{m}\left( \Omega\right) \right\}P_{1}\left( z \right)P_{2}\left( z \right)$ | (32) |

The imaginary part of $\gamma_{m}\left( \Omega\right)$ is positive. The inter-modal forward SBS process therefore leads to the attenuation of the higher-frequency pump wave, and the amplification of the lower frequency one. The analysis may be retraced in a similar manner for the case of counter-propagating stimulated acoustic waves, with the higher-frequency pump polarized along the fast $\hat{\boldsymbol{y}}$ axis (see above, and Supplementary Figure S3a, right).

*3.3 Non-reciprocal cross-polarization coupling of probe waves in inter-modal forward stimulated Brillouin scattering.*

In this section, we examine the effect of the acoustic waves stimulated by inter-modal forward SBS on a counter-propagating optical probe wave. We consider the case in which the stimulated acoustic wave is co-propagating with the two pump tones in the positive $\hat{\boldsymbol{z}}$ direction (Supplementary Figure S3a, left). Suppose that a probe of optical frequency $\omega_{\mathrm{sig}}$ is polarized along the slow $\hat{\boldsymbol{x}}$ axis and propagates in the negative $-\hat{\boldsymbol{z}}$ direction. The wavenumber of the probe equals $-k_{sig,s}={-n_{s}\omega_{\mathrm{sig}}}/c$, and we denote again its magnitude as $A_{\mathrm{sig}}$. The nonlinear polarization induced by the stimulated acoustic wave includes the following term:

| $\boldsymbol{P}_{m,+\Omega}^{\mathrm{NL}}\left( x,y,z,t \right)=\varepsilon_{0}A_{\mathrm{sig}}\left( z \right)E_{T}\left( x,y \right)\frac{1}{{4nc\rho}_{1}}Q_{\mathrm{ES}}^{\left( m \right)}H_{m}\left( \Omega\right)\tilde{P}\left( \Omega,z \right)\exp\left[ j\left( {-k}_{sig,s}+q_{m} \right)z-j\left( \omega_{\mathrm{sig}}+\Omega\right)t \right]\boldsymbol{\mu}_{m}\left( x,y \right)\left( \begin{matrix} 1 \\ 0 \end{matrix} \right)+c.c.$ | (33) |
| --- | --- |

The nonlinear polarization term may couple the input probe wave to a field component of frequency $\omega_{\mathrm{sig}}+\Omega$, co-propagating in the negative $-\hat{\boldsymbol{z}}$ direction. Efficient intra-polarization coupling along the $\hat{\boldsymbol{x}}$ axis requires that the wavenumber of the nonlinear polarization should equal ${-n_{s}\left( \omega_{\mathrm{sig}}+\Omega\right)}/c=-\left( k_{sig,s}+K_{s} \right)$. However, as seen in Eq. (33), this process is suppressed by a large wavenumber mismatch:

| ${\Delta k}_{\mathrm{mis}}=\left( -k_{sig,s}+q_{m} \right)+\left( k_{sig,s}+K_{s} \right)=q_{m}+K_{s}\approx q_{m}$ | (34) |
| --- | --- |

The mismatch ${\Delta k}_{mis}$ is on the order of 1,000 rad×m^-1^, too large for effective coupling over meters of fibre. On the other hand, cross-polarization coupling to $\hat{\boldsymbol{y}}$ polarized sideband would take place if the wavenumber of nonlinear polarization matches ${-n_{f}\left( \omega_{\mathrm{sig}}+\Omega\right)}/c=-\left( k_{sig,f}+K_{f} \right)$. Here again $-k_{sig,f}={-n_{f}\omega_{\mathrm{sig}}}/c$. The wavenumber mismatch for that process is given by:

| ${\Delta k}_{\mathrm{mis}}=\left( -k_{sig,s}+q_{m} \right)+\left( k_{sig,f}+K_{f} \right)=-\omega_{\mathrm{sig}}\left( n_{s}-n_{f} \right)/c+\omega_{p}\left( n_{s}-n_{f} \right)/c+\frac{1}{2}\Omega\left( n_{s}+n_{f} \right)/c+\Omega{n_{f}}/c=\left( \omega_{p}-\omega_{\mathrm{sig}} \right)\left( n_{s}-n_{f} \right)/c+\frac{1}{2}\Omega\left( n_{s}+{3n}_{f} \right)/c\approx\left( \omega_{p}-\omega_{\mathrm{sig}} \right)\left( n_{s}-n_{f} \right)/c+2n\Omega/c$ | (35) |
| --- | --- |

Recall that $\omega_{p}$ is the central optical frequency of the two pump waves, and $q_{m}=\omega_{p}\left( n_{s}-n_{f} \right)/c+\frac{1}{2}\Omega\left( n_{s}+n_{f} \right)/c$. The wavenumber mismatch $\Delta k$ may be set to zero, if the optical frequency of the probe is chosen above that of the pump waves with a specific offset (Supplementary Figure S3b):

| $\left( \omega_{\mathrm{sig}}-\omega_{p} \right)=\frac{2n}{n_{s}-n_{f}}\Omega\equiv{\Delta\omega}_{\mathrm{opt}}$ | (36) |
| --- | --- |


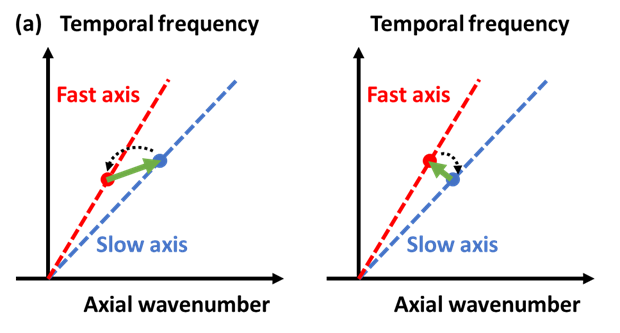

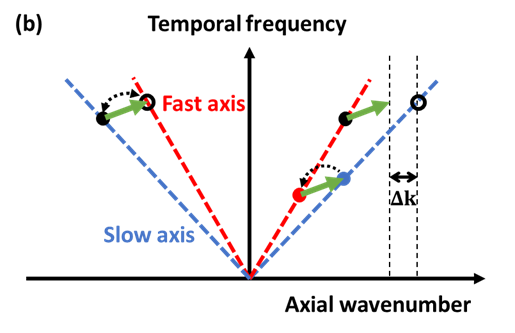


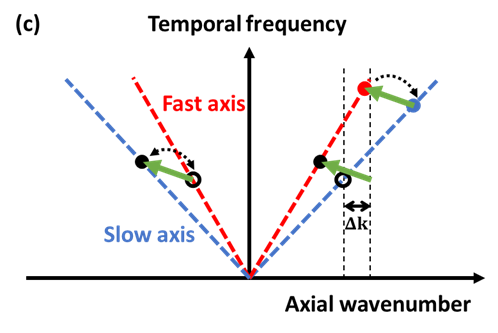


**Supplementary Figure S3.** **Inter-polarization** **forward stimulated Brillouin scattering processes in polarization maintaining fibres**. (a) – Inter-modal forward SBS. Two pump tones of orthogonal polarizations stimulate an acoustic wave. The axial wavenumber of the acoustic wave is much larger than those of intra-modal forward SBS. When the higher-frequency pump was is polarized in the slow axis, the stimulated acoustic wave is co-propagating with the two pumps (left). If the higher-frequency pump is in the fast axis instead, the acoustic wave is counter-propagating (right). (b) - Non-reciprocal cross-polarization coupling of probe waves due to inter-modal forward SBS. The acoustic wave may couple a probe wave of specific frequency that is counter-propagating with respect to the pumps to the orthogonal polarization. Cross-polarization coupling of probe light with the same frequency is prohibited in the forward direction, due to wavenumber mismatch (noted as $\Delta k$). The acoustic wave is co-propagating with the two pump tones, as in panel (a), left. (c) – Same as panel (b), for an inter-polarization forward SBS process in which the acoustic wave is counter-propagating with respect to the two pumps (see also panel (a), right).

For acoustic frequencies in the hundreds of MHz range, the optical frequency detuning $\Delta\omega$ between pumps and probe would be on THz scale, corresponding to a wavelength difference on the order of 10 nm. Since the spectrum of inter-modal forward SBS consists of multiple acoustic modes of different cut-off frequencies $\Omega_{m}$, cross-polarization coupling may be induced in several probe wavelength ranges for the same pump frequency $\omega_{p}$ through various acoustic frequencies. Following the analyses of previous sections, the optical power efficiency of cross-polarization switching due to mode $m$ at frequency $\Omega$, and with $\left( \omega_{\mathrm{sig}}-\omega_{p} \right)$ set to ${\Delta\omega}_{\mathrm{opt}}$, equals $\left| \gamma_{m}\left( \Omega\right) \right|^{2}\left| \tilde{P}\left( \Omega\right) \right|^{2}L^{2}$. Here $L$ is the length of the fibre. For the fibre length and pump power used in this work the efficiency is modest: on the order of -43 dB. When the frequency of the probe wave is not optimized, the efficiency of the process would degrade by a factor of $\mathrm{sinc}^{2}\left( {{\Delta k}_{\mathrm{mis}}L}/2 \right)=\mathrm{sinc}^{2}\left[ {\left( \omega_{\mathrm{sig}}-\omega_{p}-{\Delta\omega}_{\mathrm{opt}} \right)\left( n_{s}-n_{f} \right)L}/\left( 2c \right) \right]$. For the 22 metres-long fibre under test used in this work, the full width at half maximum of the cross-polarization coupling spectrum is 0.25 nm.

The cross-polarization coupling of probe light described above is non-reciprocal. Let us now examine the reversal of probe input and output, and consider a probe wave of frequency $\omega_{\mathrm{sig}}=\omega_{p}+{\Delta\omega}_{\mathrm{opt}}$ and $\hat{\boldsymbol{y}}$ polarization that is launched at $z=0$ and propagates in the positive $\hat{\boldsymbol{z}}$ direction alongside the pump waves. The nonlinear polarization vector at frequency $\omega_{\mathrm{sig}}+\Omega$ would be of the following form:

| $\boldsymbol{P}_{m,+\Omega}^{\mathrm{NL}}\left( x,y,z,t \right)=\varepsilon_{0}A_{\mathrm{sig}}\left( z \right)E_{T}\left( x,y \right)\frac{1}{{4nc\rho}_{1}}Q_{\mathrm{ES}}^{\left( m \right)}H_{m}\left( \Omega\right)\tilde{P}\left( \Omega,z \right)\exp\left[ j\left( k_{sig,f}+q_{m} \right)z-j\left( \omega_{\mathrm{sig}}+\Omega\right)t \right]\boldsymbol{\mu}_{m}\left( x,y \right)\left( \begin{matrix} 0 \\ 1 \end{matrix} \right)+c.c.$ | (37) |
| --- | --- |

This time, the wavenumber mismatch for cross-polarization scattering would be appreciable:

| ${\Delta k}_{\mathrm{mis}}=\left( k_{sig,f}+q_{m} \right)-\left( k_{sig,s}+K_{s} \right)=\omega_{\mathrm{sig}}\left( n_{f}-n_{s} \right)/c+\omega_{p}\left( n_{s}-n_{f} \right)/c+\frac{1}{2}\Omega\left( n_{s}+n_{f} \right)/c-\Omega{n_{s}}/c=\left( \omega_{p}-\omega_{\mathrm{sig}} \right)\left( n_{s}-n_{f} \right)/c-\frac{1}{2}\Omega\left( n_{s}-n_{f} \right)/c\approx-{2n\Omega}/c$ | (38) |
| --- | --- |

The mismatch $\left| {\Delta k}_{\mathrm{mis}} \right|$ is on the order of 10 rad×m^-1^, and it would prevent cross-polarization coupling of forward propagating probe waves over fibres that are tens of meters long (Supplementary Figure S3b).

As noted earlier, if the pump wave of the higher frequency $\omega_{p}+\frac{1}{2}\Omega$ is polarized along the fast $\hat{\boldsymbol{y}}$ axis and the lower-frequency pump along the slow $\hat{\boldsymbol{x}}$ axis, inter-polarization forward SBS would result in a backward-propagating acoustic wave. Repeating the above analysis, we find that non-reciprocal coupling may take place for an input probe wave of frequency $\omega_{\mathrm{sig}}$ that is $\hat{\boldsymbol{y}}$ polarized and propagates in the negative $-\hat{\boldsymbol{z}}$ direction (Supplementary Figure S3c). The input probe wave would be coupled to an $\hat{\boldsymbol{x}}$ polarized wave component in the same direction, that is upshifted in frequency to $\omega_{\mathrm{sig}}+\Omega$. This time, the process is wavenumber matched for a probe frequency below those of the pumps: $\omega_{\mathrm{sig}}=\omega_{p}-{\Delta\omega}_{\mathrm{opt}}$. Here too, a probe wave of the same frequency in the $+\hat{\boldsymbol{z}}$ direction would not experience cross-polarization coupling.

In summary of this section, standard PM fibres support inter-polarization forward SBS. The process leads to cross-polarization coupling of probe waves that are counter-propagating with respect to the pumps. Such coupling is non-reciprocal. This property is analogous to previous demonstrations in nano-structured fibres and photonic integrated circuits^6,9-19^, now carried over to a standard, off-the-shelf optical medium.

**Supplementary References**

1. Shelby, R. M., Levenson, M. D. & Bayer, P. W. Guided acoustic-wave Brillouin scattering. *Physical* *Review* *B* **31**, 5244-5252 (1985).
2. Biryukov, A. S., Sukharev, M. E. & Dianov, E. M. Excitation of sound waves upon propagation of laser pulses in optical fibres. *Quantum Electronics* **32**, 765-775 (2002).
3. Russell, P. S. J., Culverhouse, D. & Farahi, F. Experimental observation of forward stimulated Brillouin scattering in dual-mode single-core fibre. *Electronics Letters* **26**, 1195-1196 (1990).
4. Auld, B. A. *Acoustic* *Fields* *and* *Waves* *in* *Solids*. (New York: Wiley, 1973).
5. Wang, J. *et al*. FSBS resonances observed in a standard highly nonlinear fiber. *Optics* *Express* **19**, 5339-5349 (2011).
6. Engan, H. E. *et al*. Propagation and optical interaction of guided acoustic waves in two-mode optical fibers. *Journal of Lightwave Technology* **6**, 428-436 (1988).
7. Boyd, R. W. *Nonlinear Optics,* 3rd Edition. Academic (2008).
8. Wolff, C. *et al*. Cascaded forward Brillouin scattering to all Stokes orders. *New Journal of Physics* **19**, 023021 (2017).
9. Gertler, S. *et al*. Microwave filtering using forward Brillouin scattering in photonic-phononic emit-receive devices. *Journal of Lightwave Technology* **38**, 5248-5261 (2020).
10. Kang, M. S., Brenn, A. & Russell, P. S. J. All-optical control of gigahertz acoustic resonances by forward stimulated interpolarization scattering in a photonic crystal fiber. *Physical Review Letters* **105**, 153901 (2010).
11. Butsch, A. *et al*. CW-pumped single-pass frequency comb generation by resonant optomechanical nonlinearity in dual-nanoweb fiber. *Optica* **1**, 158-164 (2014).
12. Pang, M. *et al*. All-optical bit storage in a fibre laser by optomechanically bound states of solitons. *Nature* *Photonics* **10**, 454-458 (2016).
13. Koehler, J. R. *et al.* Resolving the mystery of milliwatt-threshold opto-mechanical self-oscillation in dual-nanoweb fiber. *APL Photonics* **1**, 056101(2016).
14. Sohn, D. B., Kim, S. & Bahl, G. Time-reversal symmetry breaking with acoustic pumping of nanophotonic circuits. *Nature* *Photonics* **12**, 91-97 (2018).
15. Otterstrom, N. T. *et al*. A silicon Brillouin laser. *Science* **360**, 1113-1116 (2018).
16. Otterstrom, N. T. *et al.* Resonantly enhanced nonreciprocal silicon Brillouin amplifier. *Optica* **6**, 1117-1123 (2019).
17. Gertler, S. *et al*. Tunable microwave-photonic filtering with high out-of-band rejection in silicon. *APL Photonics* **5**, 096103 (2020).
18. Otterstrom, N. T. *et al.* Backscatter-immune injection-locked Brillouin laser in silicon. *Physical Review Applied* **14**, 044042 (2020).
19. Kittlaus, E. A. *et al.* Electrically driven acousto-optics and broadband non-reciprocity in silicon photonics. *Nature Photonics* **15**, 43-52 (2021).
